# Supplementary material for: Intersection of Immunity, Metabolism, and Muscle Regeneration in an Autoimmune‐Prone MRL Mouse Model
Source: Adv Sci (Weinh). 2024 Jan 8;11(11):2306961. doi: 10.1002/advs.202306961 (PMC10953568; doi:10.1002/advs.202306961)
Supplement: Supplementary file 1 — Supporting Information [file ADVS-11-2306961-s001.pdf]

ADVANCED SCIENCE
Open Access

Supporting Information

for *Adv. Sci.*, DOI 10.1002/advs.202306961

Intersection of Immunity, Metabolism, and Muscle Regeneration in an Autoimmune-Prone MRL Mouse Model

*Tran B. Ngo, Aditya Josyula, Sabrina DeStefano, Daphna Fertil, Mondreakest Faust, Ravi Lokwani and Kaitlyn Sadtler**

Supporting Information

Intersection of Immunity, Metabolism, and Muscle Regeneration in an Autoimmune-Prone MRL Mouse Model

Tran B. Ngo, Aditya Josyula, Sabrina DeStefano, Daphna Fertil, Mondreakest Faust, Ravi Lokwani, Kaitlyn Sadtler

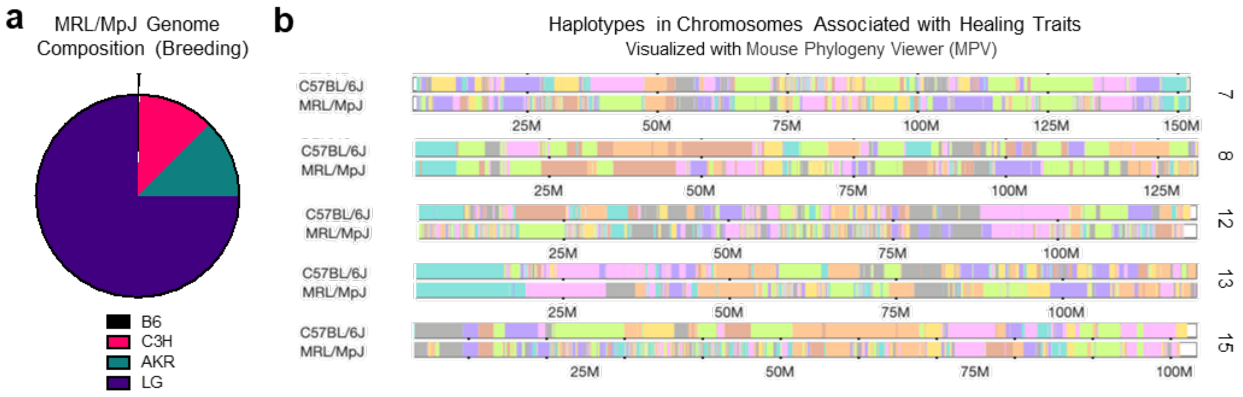

Supplementary Figure 1 | (a) Genomic composition of MRL/MpJ mouse from parent strains **(b)** Haplotype display on chromosomes 7, 8, 12, 13, and 15 from Mouse Phylogeny Viewer JR Wang, F Pardo-Manuel de Villena, and L McMillan. *Comparative analysis and visualization of multiple collinear genomes*. BMC Bioinformatics, 2012.

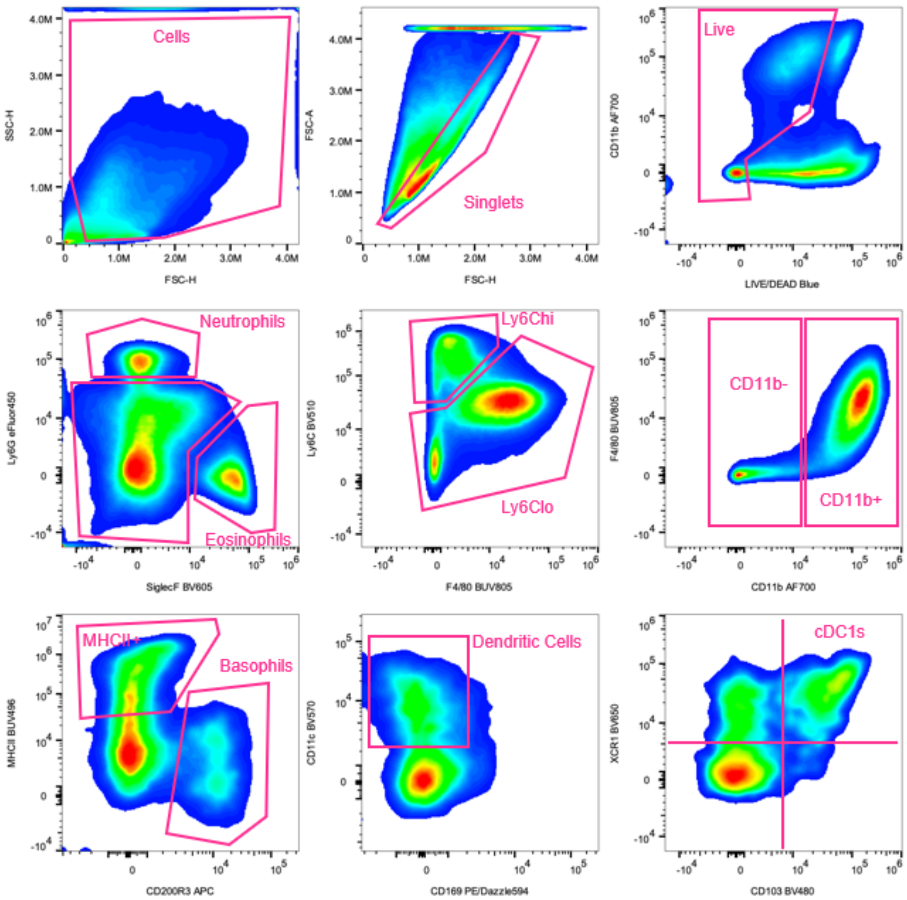

Supplementary Figure 2 | Gating Strategy for myeloid flow cytometry panel. Top row = control injury at 21 days post-op. The bottom two rows = Control, ECM_{tx}, and PE_{tx} concatenated (21 days post-injury). C57BL/6 mouse.

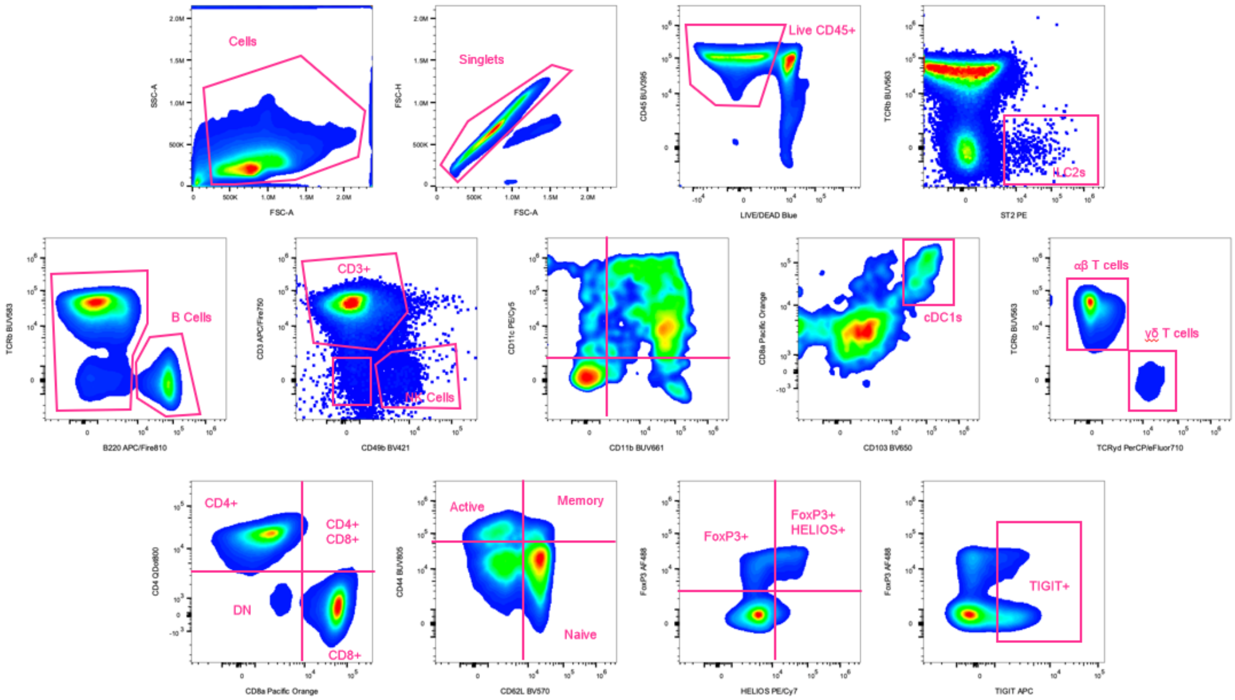

Supplementary Figure 3 | Gating Strategy for lymphoid flow cytometry panel. Control injury at 21 days post-injury. Inguinal lymph node from C57BL/6 mouse.

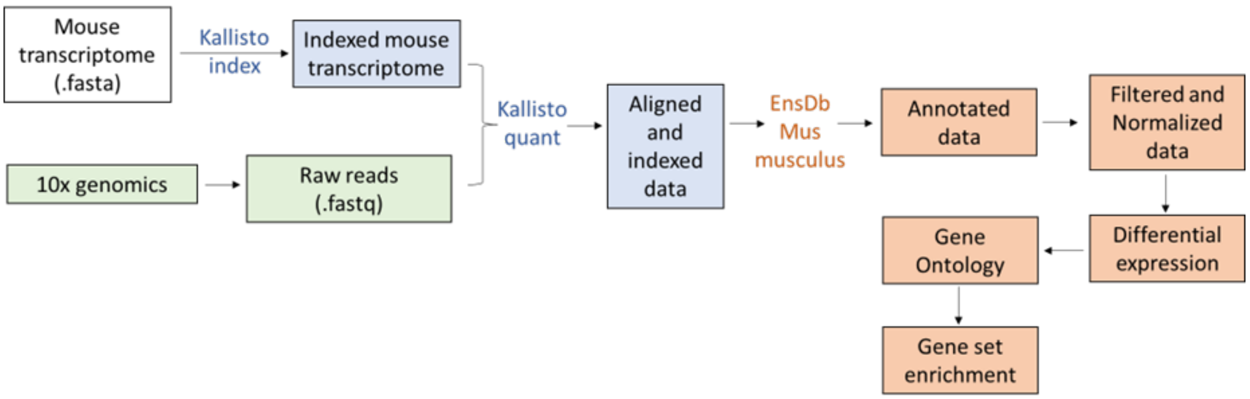

Supplementary Figure 4 | Schematic of bulk RNA sequencing data analysis workflow. Panels in green represent data obtained directly from Azenta Genewiz. Panels in blue represent data analyzed using the command line program Kallisto. Panels in red represent data analyzed using R (version 4.2.1 'Funny-looking kid').

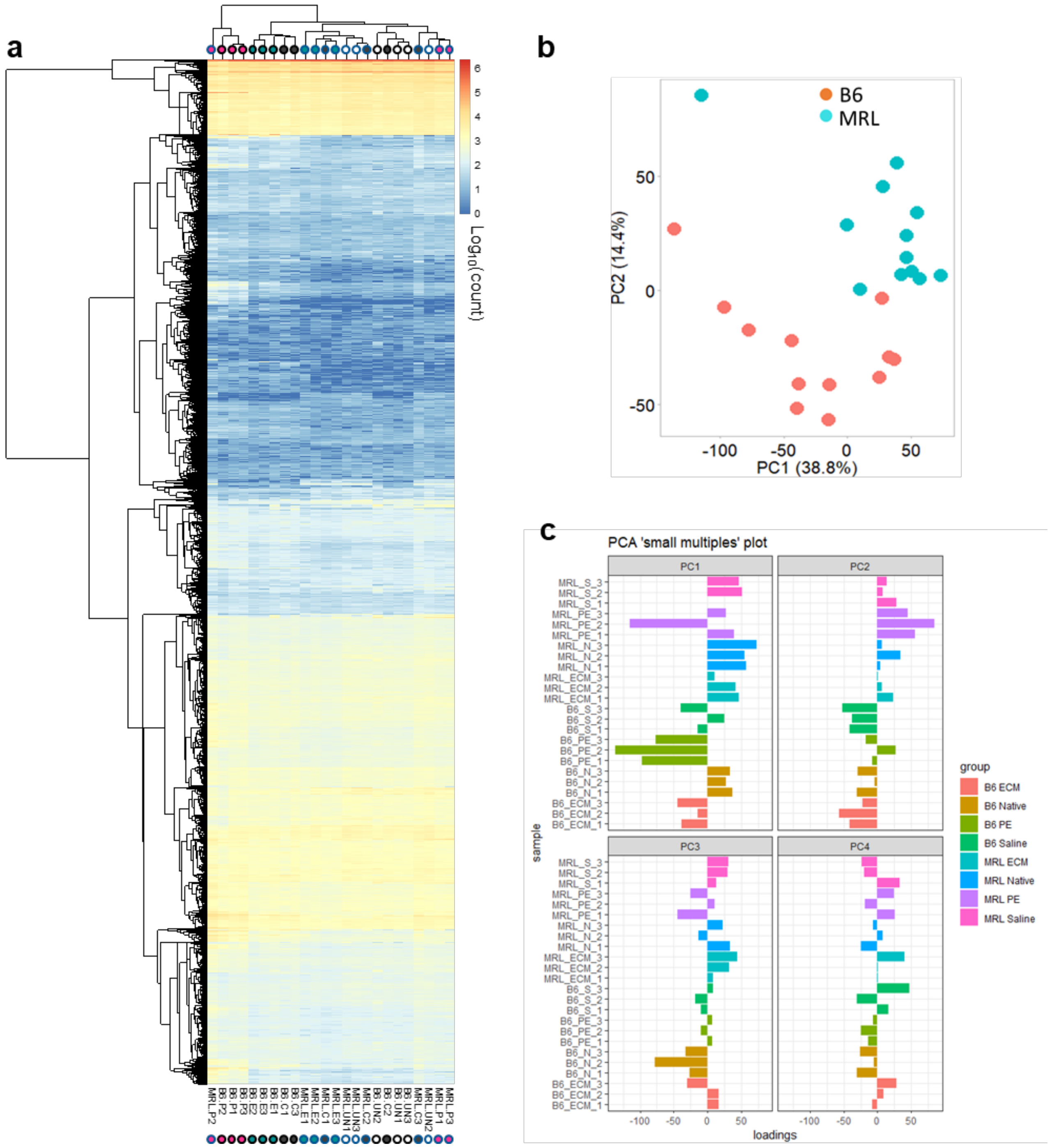

Supplementary Figure 5 | (**a**) Heatmap of differentially expressed genes across all samples with dendrograms representing clustering performed by row (**left**) and column (**top**). (**b**) Principal component analysis of all samples with dot plot showing variation represented by PC1 and PC2 (top) and (**c**) small multiples plot of principal components 1-4. The color gradient scale bar in (**a**) represents Log_{10}(CPM).

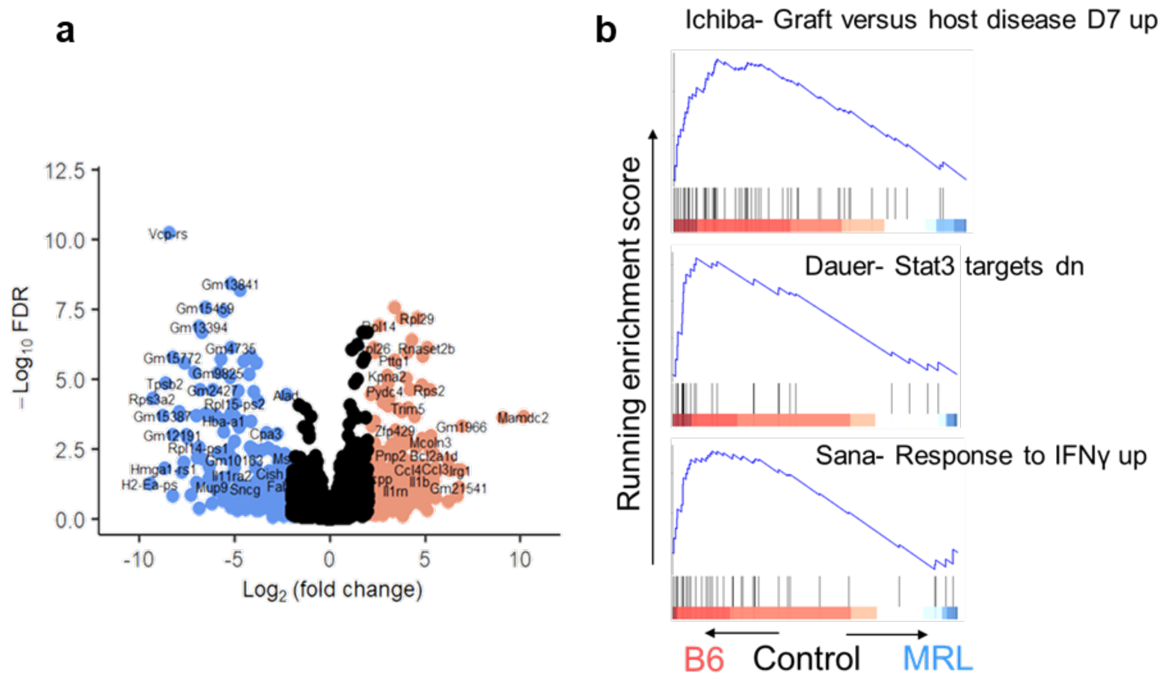

Supplementary Figure 6 | (**a**) Volcano plot of differentially expressed genes in the control injured group in B6 versus MRL mice. (**b**) GSEA of top 250 differentially expressed genes from (**a**) with relevant gene sets displayed.

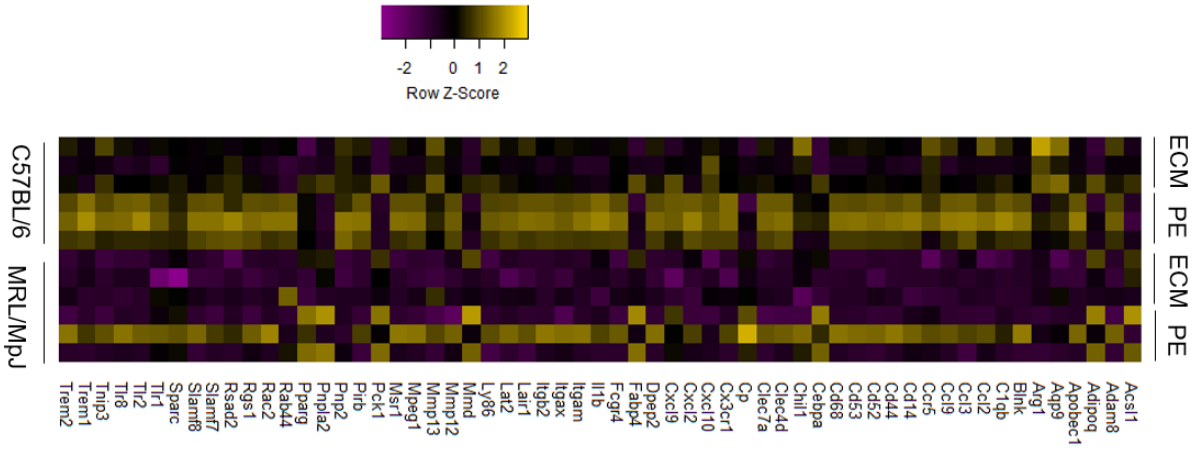

Supplementary Figure 7 | Heatmap of genes potentiated by PE_{tx} compared to ECM_{tx} in C57BL/6 and MRL/MpJ mouse strain.

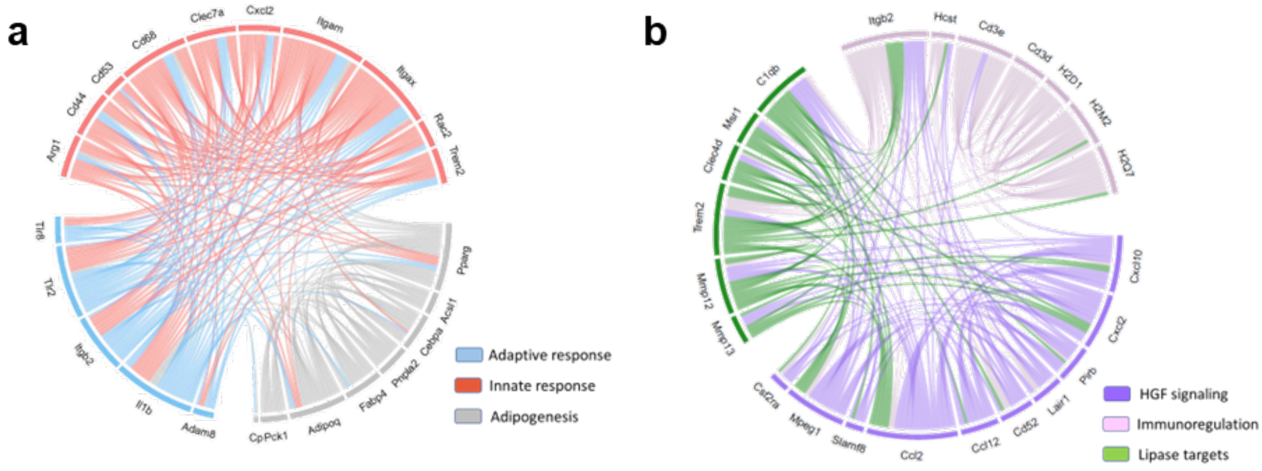

Supplementary Figure 8 | Chord diagrams of protein-protein interactions sourced from the string protein database. Interactions of leading-edge markers from gene sets enriched in (**a**) PE_{tx} and (**b**) ECM_{tx} obtained from top 250 differentially expressed genes across B6 and MRL mice.

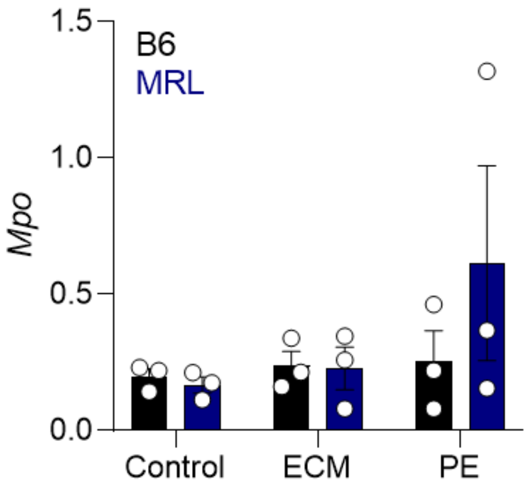

Supplementary Figure 9 | MPO gene expression of a neutrophil marker in saline control, ECM-treated, and PE-treated groups in C57BL/6 and MRL/MpJ mice.

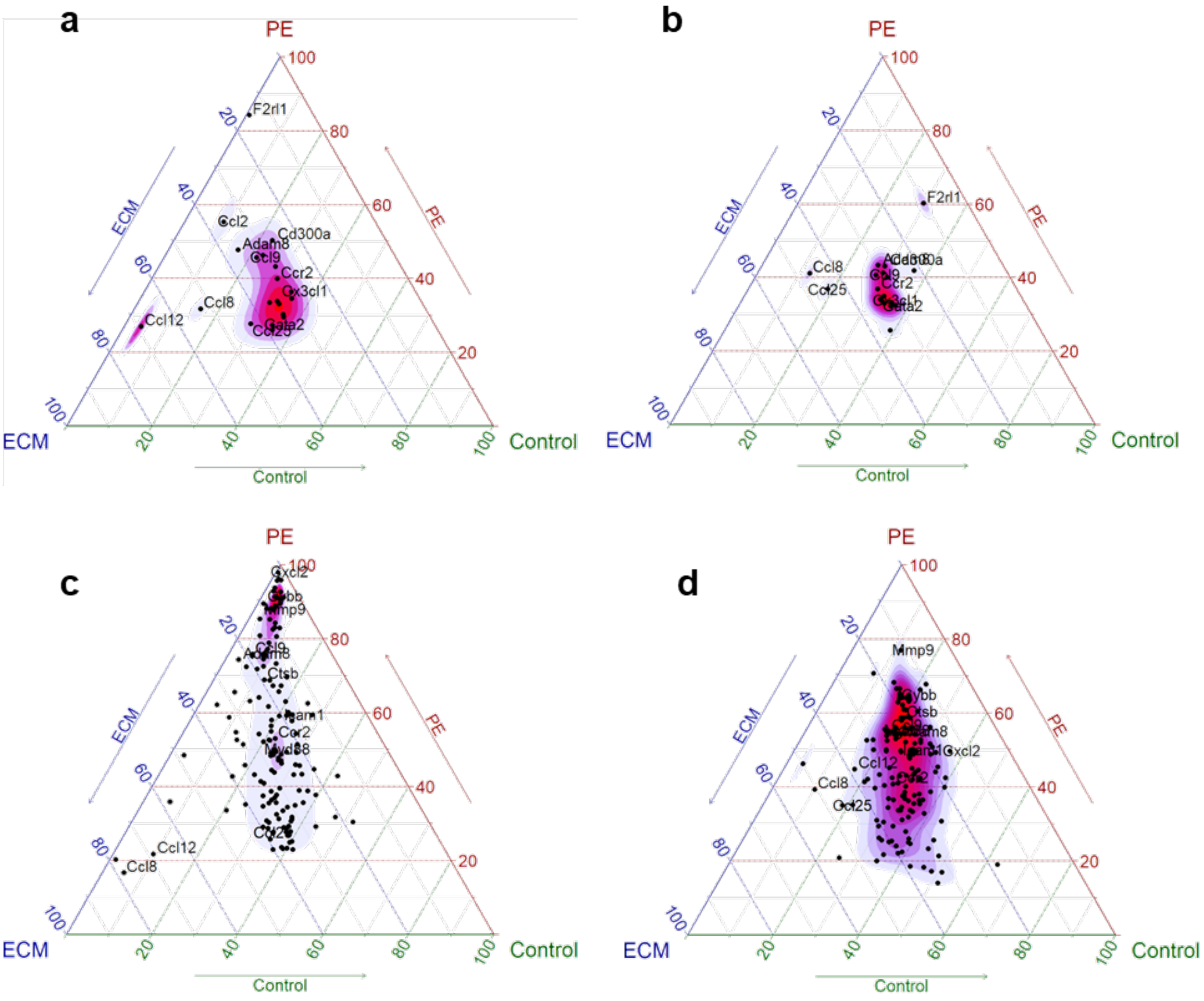

Supplementary Figure 10 | Ternary plots showing rescaled normalized log_2(CPM) gene expression of eosinophil markers in (**a**) C57BL/6 and (**b**) MRL/MpJ mice. Similarly, neutrophil markers in (**c**) C57BL/6 and (**d**) MRL/MpJ mice. All data points represent individual genes' average log2(CPM) values at 21 days post VML and material implantation. Eosinophil markers were subset from the gene set "GOBP: Eosinophil mediated immunity" and neutrophil markers from "Biocarta: Neutrophil degranulation."

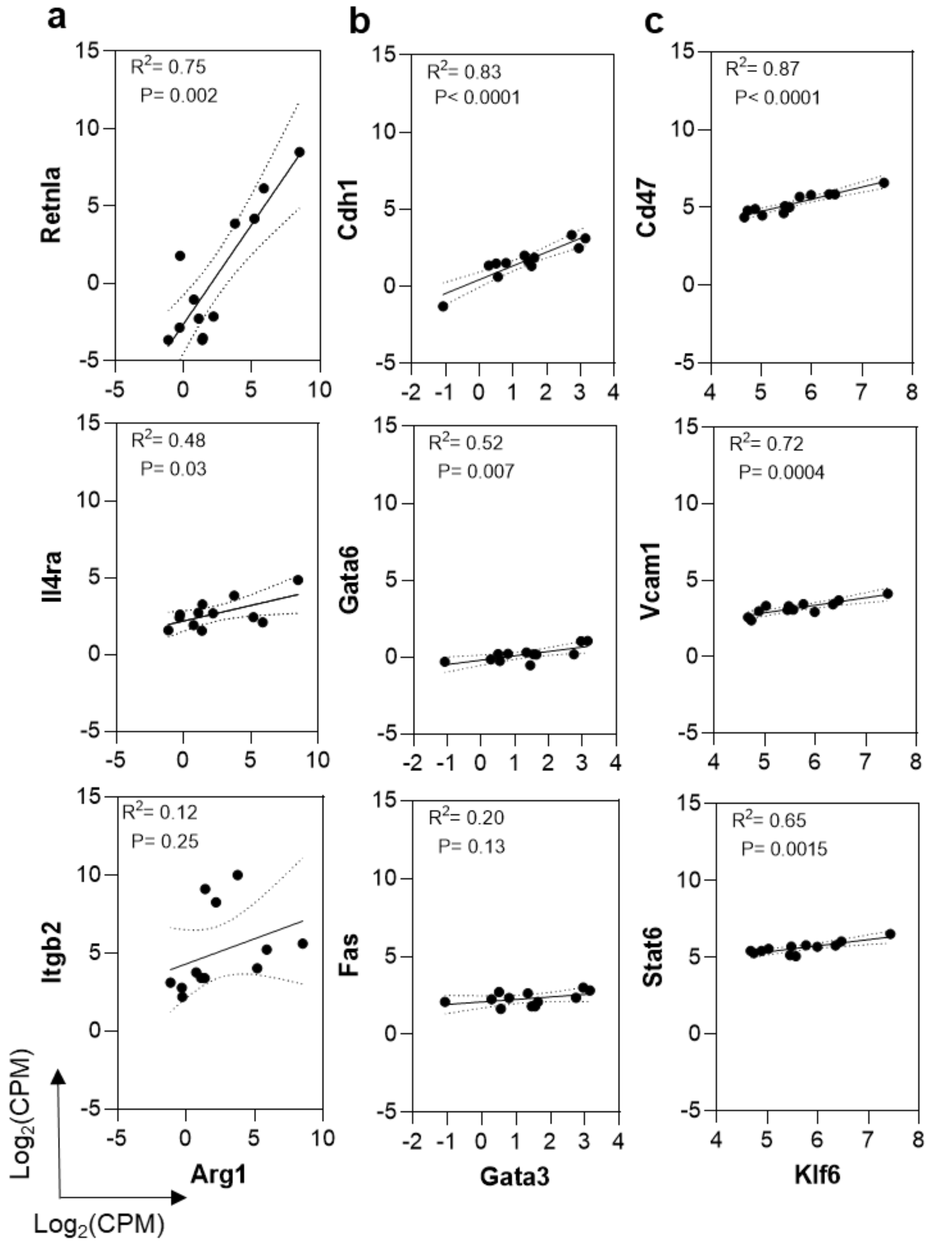

Supplementary Figure 11 | The M2 macrophage markers (**a**) *Arg1,* (**b**) *Gata3,* and (**c**) *Klf6* were strongly co-expressed ($R^2 \geq 0.75$) with *Retnla, Cdh1,* and *Cd47,* respectively, in all B6 mice across all treatments.

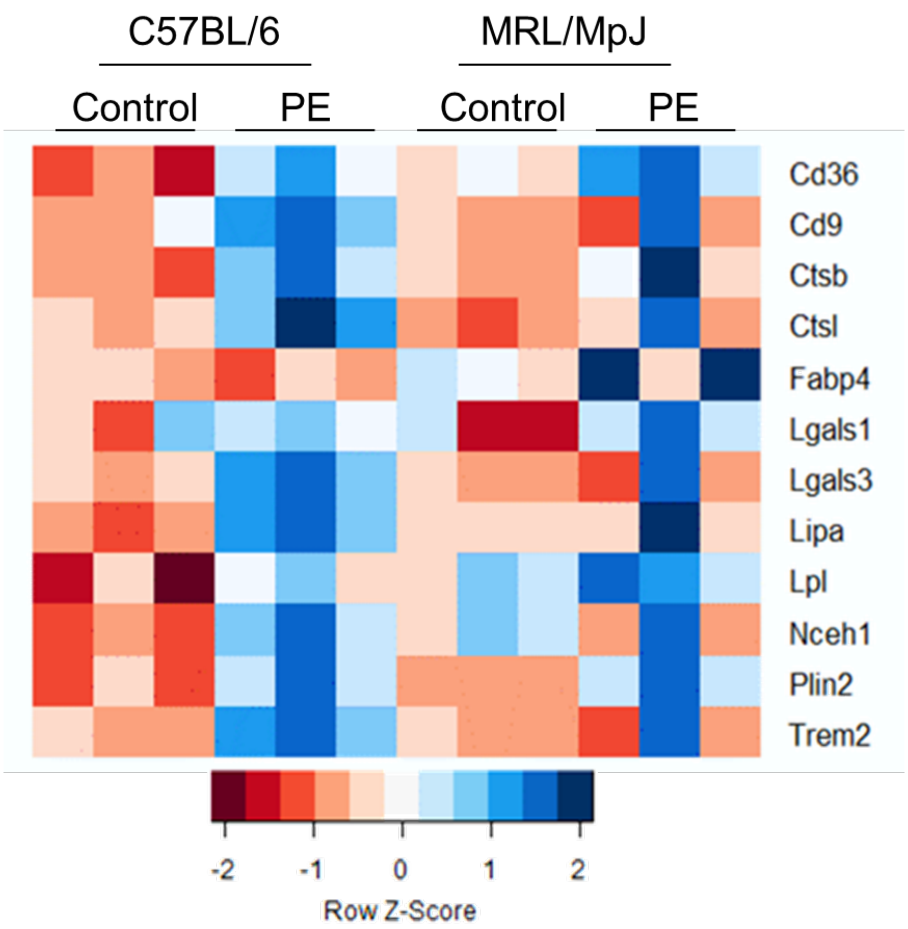

Supplementary Figure 12 | Heatmap of genes comprising lipid-associated macrophage signature enriched in mice that received PE_{tx} compared to saline controls.

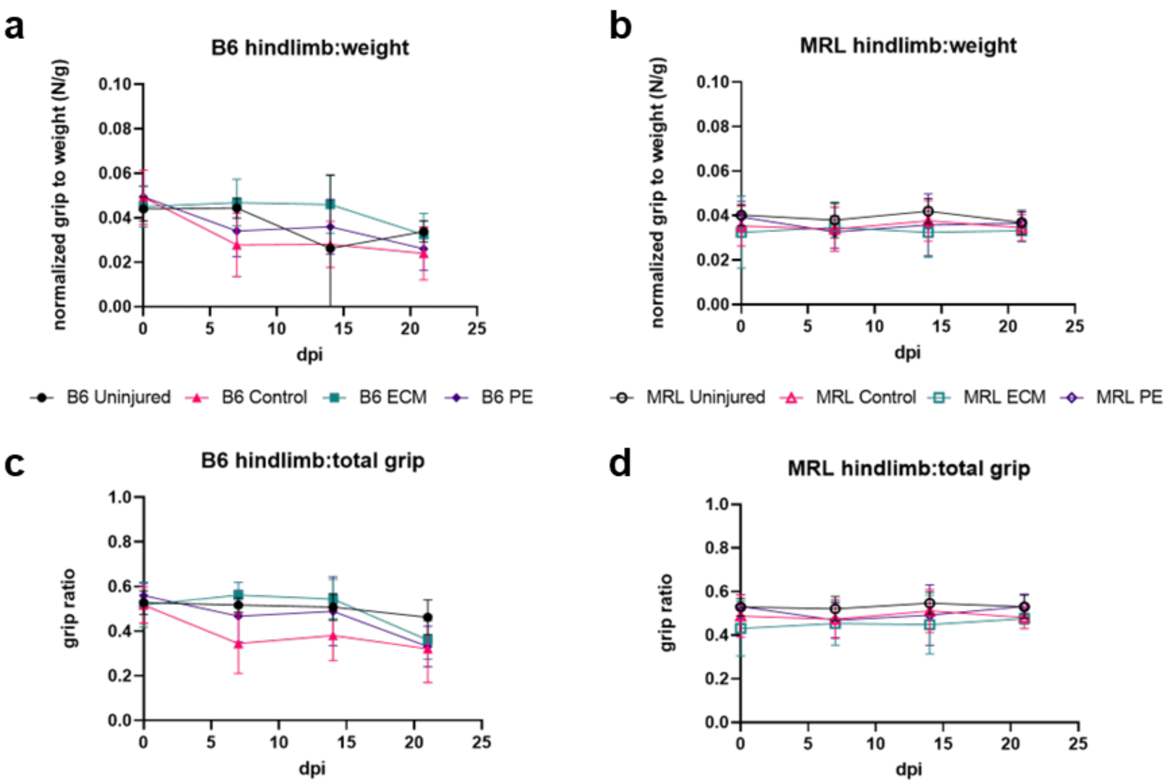

Supplementary Figure 13 | Normalized grip strength of B6 versus MRL mice pre- and post-injury with different material implantations. (a) and **(b)** Hindlimb grip strength measurements normalized to body weight at pre-injury and 7-, 14-, and 21-days post-injury of B6 versus MRL mice, respectively. **(c)** and **(d)** Hindlimb grip strength measurements normalized to total 4-limbs strength at pre-injury and 7-, 14-, and 21-days post-injury of B6 versus MRL mice, respectively. dpi = days post-injury. Black = uninjured, pink = control injury, teal = ECM_{tx}, purple = PE_{tx}. Data are means ± SEM.

Sample ID	Barcode Sequence	# Reads	Yield (Mbases)	Mean Quality Score	% Bases >= 30
B6-PE-2	GTAGAGGA+ CTTCGCCT	23341152	7002	35.55	91.8
B6-Uninjured-3	GCTCATGA+T CAGAGCC	30067196	9020	35.58	91.93
B6-PE-1	GTAGAGGA+ TCAGAGCC	32820587	9846	35.52	91.65
B6-Uninjured-1	GCTCATGA+ GCCTCTAT	30468441	9141	35.55	91.77
B6-Uninjured-2	GCTCATGA+ AGGATAGG	29612058	8884	35.59	91.99
B6-PE-3	GTAGAGGA+ TAAGATTA	33383202	10015	35.6	92.06
MRL-ECM-1	GCTCATGA+ CTTCGCCT	26235642	7871	35.62	92.11
MRL-ECM-2	GCTCATGA+T AAGATTA	31055947	9317	35.67	92.4
MRL-PE-1	GCTCATGA+ GTCAGTAC	30772510	9232	35.58	91.96
MRL-Uninjured-3	ATCTCAGG+ GTCAGTAC	39984070	11995	35.53	91.68
B6-Saline-2	GTAGAGGA+ GTCAGTAC	34568555	10371	35.5	91.56
MRL-PE-2	ATCTCAGG+ AGGCTATA	35226447	10568	35.47	91.42
MRL-Uninjured-2	ATCTCAGG+ ACGTCCTG	41136587	12341	35.65	92.29
MRL-Uninjured-1	ATCTCAGG+T AAGATTA	36836367	11051	35.62	92.11
B6-ECM-2	GTAGAGGA+ GCCTCTAT	27006422	8102	35.5	91.58
MRL-ECM-3	GCTCATGA+ ACGTCCTG	25466670	7640	35.62	92.21
B6-Saline-1	GTAGAGGA+ ACGTCCTG	29484030	8845	35.61	92.11
B6-ECM-3	GTAGAGGA+ AGGATAGG	31221810	9367	35.56	91.88
MRL-Saline-2	ATCTCAGG+T CAGAGCC	34998055	10499	35.54	91.71
B6-Saline-3	GCTCATGA+ AGGCTATA	25044049	7513	35.53	91.73
MRL-PE-3	ATCTCAGG+ GCCTCTAT	30143707	9043	35.52	91.61

B6-ECM-1	GTAGAGGA+ AGGCTATA	28875619	8663	35.51	91.63
MRL-Saline-1	ATCTCAGG+ AGGATAGG	36356638	10907	35.51	91.59
MRL-Saline-3	ATCTCAGG+ CTTCGCCT	24880999	7464	35.6	92

Supplementary Table 1 | Bulk RNA sequencing quality control metrics for all 24 samples in this study, including mean quality score and total number of reads per sample.

Name used in figure	Full gene set name
Adipogenesis	Burton Adipogenesis
Adaptive Immune Response	Reactome-Adaptive Immune System
Innate Immune Response	Reactome- Innate Immune System
HGF Signaling	Rutella-Response to HGF Up
Lipase targets	Lian Lipa Targets 3M
IL4 singlaing	GOBP: Interleukin 4 mediated signaling pathway
Neutrophils	Reactome- Neutrophil Degranulation
TNF-p38	Phong- TNF response via p38 complete
Lipase targets	Lian Lipa Targets 3M
Ribosome	KEGG- Ribosome
Fibrosis	WP-Overview of proinflammatory and profibrotic mediators
Steroid	Reactome- Metabolism of Steroids
Lipids	Reactome- Metabolism of Lipids
Energy	Reactome- Integration of energy metabolism
Mitochondria	Mootha Mitochondria
Pyruvate	Reactome- Pyruvate Metabolism
Cytokine receptor	KEGG- Cytokine-Cytokine Receptor Interaction
M2 Macrophage	Coates- Macrophage M1 vs M2

Supplementary Table 2 | Full Gene set names displayed in GSEA plots. Gene set names in figures were abridged for brevity.

Supplementary Data 1 | Excel spreadsheet with the differential gene expression (DEG) statistics for all genes and all comparisons made.

Supplementary Methods | PDF document with code used to analyze bulk RNA sequencing data. See subsequent pages of this document.

Ectopic adipogenesis in response to injury and material implantation in an autoimmune mouse model

Tran Ngo, Aditya Josyula, Sabrina DeStefano, Daphna Fertil,
Mondreakest Faust, Ravi Lokwani, Kaitlyn Sadtler

produced on 2023-09-20

Contents

1 Background **2**

2 Read mapping from raw fastq files using kallisto **2**

3 R packages used for analysis **4**

4 Gene Annotation **4**

5 Data filtering and normalization **5**

6 Data Analysis **7**

6.1 PCA plot . . . 7

6.2 Volcano plot- Differential expression analysis: B6 vs MRL Saline . . . 8

6.3 Heatmap: Top 50 differentially expressed genes- B6 vs MRL Uninjured . . . 11

6.4 Gene Ontology and Pathway Assignment: Saline . . . 11

6.5 Gene Set Enrichment Analysis- B6 vs MRL: Saline . . . 12

6.6 Gene Set Enrichment Analysis- B6 vs MRL: PE . . . 13

6.7 ImmQuant scores and gene signatures correlation plot: PE . . . 14

6.8 Heatmap of differentially expressed genes: ECM vs PE . . . 15

6.9 Gene Set Enrichment Analysis- B6 vs MRL: ECM . . . 16

6.10 ImmQuant scores and gene signatures correlation plot: ECM . . . 17

6.11 Interaction plot- PE: Immune Cell Targets and Adipogenesis . . . 18

6.12 Interaction plot- ECM:HGF response and Eosinophil gene set . . . 19

6.13 Ternary plot: M1 vs M2 : Coates macrophage gene set . . . 21

6.14 Ternary plot: Neutrophil gene set . . . 24

6.15 Ternary plot: Eosinophils gene set . . . 27

1 Background

Murphy roths large (MRL/MpJ) mice are known to regenerate skin and skeletal muscle wounds without scar formation but are also susceptible to metabolic dysregulation and obesity. How biomaterials influence immune cell recruitment and immune-adipocyte interaction after muscle injury in MRL/MpJ mice is yet to be delineated. To investigate this, we performed volumetric muscle loss surgeries (VML) in C57BL/6 (B6) and MRL/MpJ mice and evaluated immune responses to injury, regenerative (decellularized extracellular matrix, ECM) and fibrotic (polyethylene, PE) material implants using multiparametric flow cytometry, RNA sequencing, and histopathology. In MRL/MpJ but not B6 mice, there was white fat deposition at the site of VML. This was correlated with enrichment of the adipogenesis gene sets including Adipoq, Cebpa, Pparg and Fabp4. In addition, we observed that F4/80- CD68+ macrophages were significantly more abundant in MRL/MpJ mice at 3 weeks post-injury in PE and control groups. Furthermore, in MRL/MpJ mice, the lipid associated macrophage gene signature comprising Trem2, Ly6c2, Cd9, Cd63, Lyz2 were highly potentiated by muscle injury as well as PE implantation. In contrast, ECM implantation produced a largely SiglecF+ eosinophilic response (~60% of CD45+ cells) and partially suppressed CD68+ macrophage abundance. Furthermore, ECM potentiated expression of the pro-regenerative Arg1, Chil3, Retnla and Gata3 in both B6 and MRL/MpJ. In PE groups, Ly6G+ neutrophils were the second most abundant cell type (~ 25% of CD45+ cells) which was corroborated by increased abundance of Vav1, Ccl2, Adam8 and Rac2. Taken together, our results suggest a potential mechanistic role for CD68+ macrophages in promoting adipogenesis after VML.

---

2 Read mapping from raw fastq files using kallisto

```
# Building an index file
kallisto index -i Mus_musculus.GRCm39.cdna.all.index Mus_musculus.GRCm39.cdna.all.fa

# Mapping reads to the index file

kallisto quant -i Mus_musculus.GRCm39.cdna.all.index -o B6_ECM_1 -t8 \
rB6-1-E1_R1_001.fastq.gz rB6-1-E1_R2_001.fastq.gz &> B6_ECM_1.log

kallisto quant -i Mus_musculus.GRCm39.cdna.all.index -o B6_ECM_2 -t8 \
rB6-2-E2_R1_001.fastq.gz rB6-2-E2_R2_001.fastq.gz &> B6_ECM_2.log

kallisto quant -i Mus_musculus.GRCm39.cdna.all.index -o B6_ECM_3 -t8 \
rB6-3-E3_R1_001.fastq.gz rB6-3-E3_R2_001.fastq.gz &> B6_ECM_3.log

kallisto quant -i Mus_musculus.GRCm39.cdna.all.index -o B6_PE_1 -t8 \
rB6-4-P1_R1_001.fastq.gz rB6-4-P1_R2_001.fastq.gz &> B6_PE_1.log

kallisto quant -i Mus_musculus.GRCm39.cdna.all.index -o B6_PE_2 -t8 \
rB6-5-P2_R1_001.fastq.gz rB6-5-P2_R2_001.fastq.gz &> B6_PE_2.log

kallisto quant -i Mus_musculus.GRCm39.cdna.all.index -o B6_PE_3 -t8 \
rB6-6-P3_R1_001.fastq.gz rB6-6-P3_R2_001.fastq.gz &> B6_PE_3.log

kallisto quant -i Mus_musculus.GRCm39.cdna.all.index -o B6_S_1 -t8 \
rB6-7-S1_R1_001.fastq.gz rB6-7-S1_R2_001.fastq.gz &> B6_S_1.log
```

```
kallisto quant -i Mus_musculus.GRCm39.cdna.all.index -o B6_S_2 -t8 \
rB6-8-S2_R1_001.fastq.gz rB6-8-S2_R2_001.fastq.gz &> B6_S_2.log

kallisto quant -i Mus_musculus.GRCm39.cdna.all.index -o B6_S_3 -t8 \
rB6-9-S3_R1_001.fastq.gz rB6-9-S3_R2_001.fastq.gz &> B6_S_3.log

kallisto quant -i Mus_musculus.GRCm39.cdna.all.index -o B6_N_1 -t8 \
rB6-10-N1_R1_001.fastq.gz rB6-10-N1_R2_001.fastq.gz &> B6_N_1.log

kallisto quant -i Mus_musculus.GRCm39.cdna.all.index -o B6_N_2 -t8 \
rB6-11-N2_R1_001.fastq.gz rB6-11-N2_R2_001.fastq.gz &> B6_N_2.log

kallisto quant -i Mus_musculus.GRCm39.cdna.all.index -o B6_N_3 -t8 \
rB6-12-N3_R1_001.fastq.gz rB6-12-N3_R2_001.fastq.gz &> B6_N_3.log


kallisto quant -i Mus_musculus.GRCm39.cdna.all.index -o MRL_ECM_1 -t8 \
rMRL-1-E1_R1_001.fastq.gz rMRL-1-E1_R2_001.fastq.gz &> MRL_ECM_1.log

kallisto quant -i Mus_musculus.GRCm39.cdna.all.index -o MRL_ECM_2 -t8 \
rMRL-2-E2_R1_001.fastq.gz rMRL-2-E2_R2_001.fastq.gz &> MRL_ECM_2.log

kallisto quant -i Mus_musculus.GRCm39.cdna.all.index -o MRL_ECM_3 -t8 \
rMRL-3-E3_R1_001.fastq.gz rMRL-3-E3_R2_001.fastq.gz &> MRL_ECM_3.log

kallisto quant -i Mus_musculus.GRCm39.cdna.all.index -o MRL_PE_1 -t8 \
rMRL-1-P1_R1_001.fastq.gz rMRL-1-P1_R2_001.fastq.gz &> MRL_PE_1.log

kallisto quant -i Mus_musculus.GRCm39.cdna.all.index -o MRL_PE_2 -t8 \
rMRL-2-P2_R1_001.fastq.gz rMRL-2-P2_R2_001.fastq.gz &> MRL_PE_2.log

kallisto quant -i Mus_musculus.GRCm39.cdna.all.index -o MRL_PE_3 -t8 \
rMRL-3-P3_R1_001.fastq.gz rMRL-3-P3_R2_001.fastq.gz &> MRL_PE_3.log

kallisto quant -i Mus_musculus.GRCm39.cdna.all.index -o MRL_S_1 -t8 \
rMRL-1-S1_R1_001.fastq.gz rMRL-1-S1_R2_001.fastq.gz &> MRL_S_1.log

kallisto quant -i Mus_musculus.GRCm39.cdna.all.index -o MRL_S_2 -t8 \
rMRL-2-S2_R1_001.fastq.gz rMRL-2-S2_R2_001.fastq.gz &> MRL_S_2.log

kallisto quant -i Mus_musculus.GRCm39.cdna.all.index -o MRL_S_3 -t8 \
rMRL-3-S3_R1_001.fastq.gz rMRL-3-S3_R2_001.fastq.gz &> MRL_S_3.log

kallisto quant -i Mus_musculus.GRCm39.cdna.all.index -o MRL_N_1 -t8 \
rMRL-10-N1_R1_001.fastq.gz rMRL-10-N1_R2_001.fastq.gz &> MRL_N_1.log

kallisto quant -i Mus_musculus.GRCm39.cdna.all.index -o MRL_N_2 -t8 \
rMRL-11-N2_R1_001.fastq.gz rMRL-11-N2_R2_001.fastq.gz &> MRL_N_2.log

kallisto quant -i Mus_musculus.GRCm39.cdna.all.index -o MRL_N_3 -t8 \
rMRL-12-N3_R1_001.fastq.gz rMRL-12-N3_R2_001.fastq.gz &> MRL_N_3.log
```

3 R packages used for analysis

```
library(tidyverse)
library(tximport)
library(ensembldb)
library(EnsDb.Mmusculus.v79)
library(edgeR)
library(matrixStats)
library(cowplot)
library(ggplot2)
library(DT)
library(gt)
library(limma)
library(EnhancedVolcano)
library(gplots)
library(GSEABase)
library(Biobase)
library(GSVA)
library(gprofiler2)
library(clusterProfiler)
library(msigdbr)
library(enrichplot)
library(corrplot)
library(RColorBrewer)
library(circlize)
library(dplyr)
library(pheatmap)
```

---

4 Gene Annotation

```
targets <- read_tsv("studydesign.txt")
path <- file.path(targets$sample, "abundance.tsv")
Tx <- transcripts(EnsDb.Mmusculus.v79, columns=c("tx_id", "gene_name"))
Tx <- as_tibble(Tx)
Tx <- dplyr::rename(Tx, target_id = tx_id)
Tx <- dplyr::select(Tx, "target_id", "gene_name")
Txi_gene <- tximport(path,
                     type = "kallisto",
                     tx2gene = Tx,
                     txOut = FALSE,
                     countsFromAbundance = "lengthScaledTPM",
                     ignoreTxVersion = TRUE)
```

---

5 Data filtering and normalization

```
sampleLabels <- targets$sample
myDGEList <- DGEList(Txi_gene$counts)
log2.cpm <- cpm(myDGEList, log=TRUE)

log2.cpm.df <- as_tibble(log2.cpm, rownames = "geneID")
colnames(log2.cpm.df) <- c("geneID", sampleLabels)
log2.cpm.df.pivot <- pivot_longer(log2.cpm.df,
                                  cols = B6_ECM_1:MRL_S_3,
                                  names_to = "samples",
                                  values_to = "expression")

p1 <- ggplot(log2.cpm.df.pivot) +
  aes(x=samples, y=expression, fill=samples) +
  geom_violin(trim = FALSE, show.legend = FALSE) +
  stat_summary(fun = "median",
               geom = "point",
               shape = 95,
               size = 10,
               color = "black",
               show.legend = FALSE) +
  labs(y="log2 expression", x = "sample",
       title="Log2 Counts per Million (CPM)",
       subtitle="unfiltered, non-normalized",
       caption=paste0("produced on ", Sys.time())) +
  theme_bw()+
  theme(axis.text.x=element_text(angle=90))

cpm <- cpm(myDGEList)
keepers <- rowSums(cpm>1)>=3
myDGEList.filtered <- myDGEList[keepers,]

log2.cpm.filtered <- cpm(myDGEList.filtered, log=TRUE)
log2.cpm.filtered.df <- as_tibble(log2.cpm.filtered, rownames = "geneID")
colnames(log2.cpm.filtered.df) <- c("geneID", sampleLabels)
log2.cpm.filtered.df.pivot <- pivot_longer(log2.cpm.filtered.df,
                                           cols = B6_ECM_1:MRL_S_3,
                                           names_to = "samples",
                                           values_to = "expression")

p2 <- ggplot(log2.cpm.filtered.df.pivot) +
  aes(x=samples, y=expression, fill=samples) +
  geom_violin(trim = FALSE, show.legend = FALSE) +
  stat_summary(fun = "median",
               geom = "point",
               shape = 95,
               size = 10,
               color = "black",
               show.legend = FALSE) +
  labs(y="log2 expression", x = "sample",
       title="Log2 Counts per Million (CPM)",
       subtitle="filtered, non-normalized",
```

```
       caption=paste0("produced on ", Sys.time()))+
  theme_bw()+
  theme(axis.text.x=element_text(angle=90))

myDGEList.filtered.norm <- calcNormFactors(myDGEList.filtered, method = "TMM")
log2.cpm.filtered.norm <- cpm(myDGEList.filtered.norm, log=TRUE)
log2.cpm.filtered.norm.df <- as_tibble(log2.cpm.filtered.norm, rownames = "geneID")
colnames(log2.cpm.filtered.norm.df) <- c("geneID", sampleLabels)
log2.cpm.filtered.norm.df.pivot <- pivot_longer(log2.cpm.filtered.norm.df,
                                               cols = B6_ECM_1:MRL_S_3,
                                               names_to = "samples",
                                               values_to = "expression")

p3 <- ggplot(log2.cpm.filtered.norm.df.pivot) +
  aes(x=samples, y=expression, fill=samples) +
  geom_violin(trim = FALSE, show.legend = FALSE) +
  stat_summary(fun = "median",
               geom = "point",
               shape = 95,
               size = 10,
               color = "black",
               show.legend = FALSE) +
  labs(y="log2 expression", x = "sample",
       title="Log2 Counts per Million (CPM)",
       subtitle="filtered, TMM normalized",
       caption=paste0("produced on ", Sys.time()))+
  theme_bw()+
    theme(axis.text.x=element_text(angle=90))

plot_grid(p1, p2, p3, labels = c('A', 'B', 'C'), label_size = 12)
```

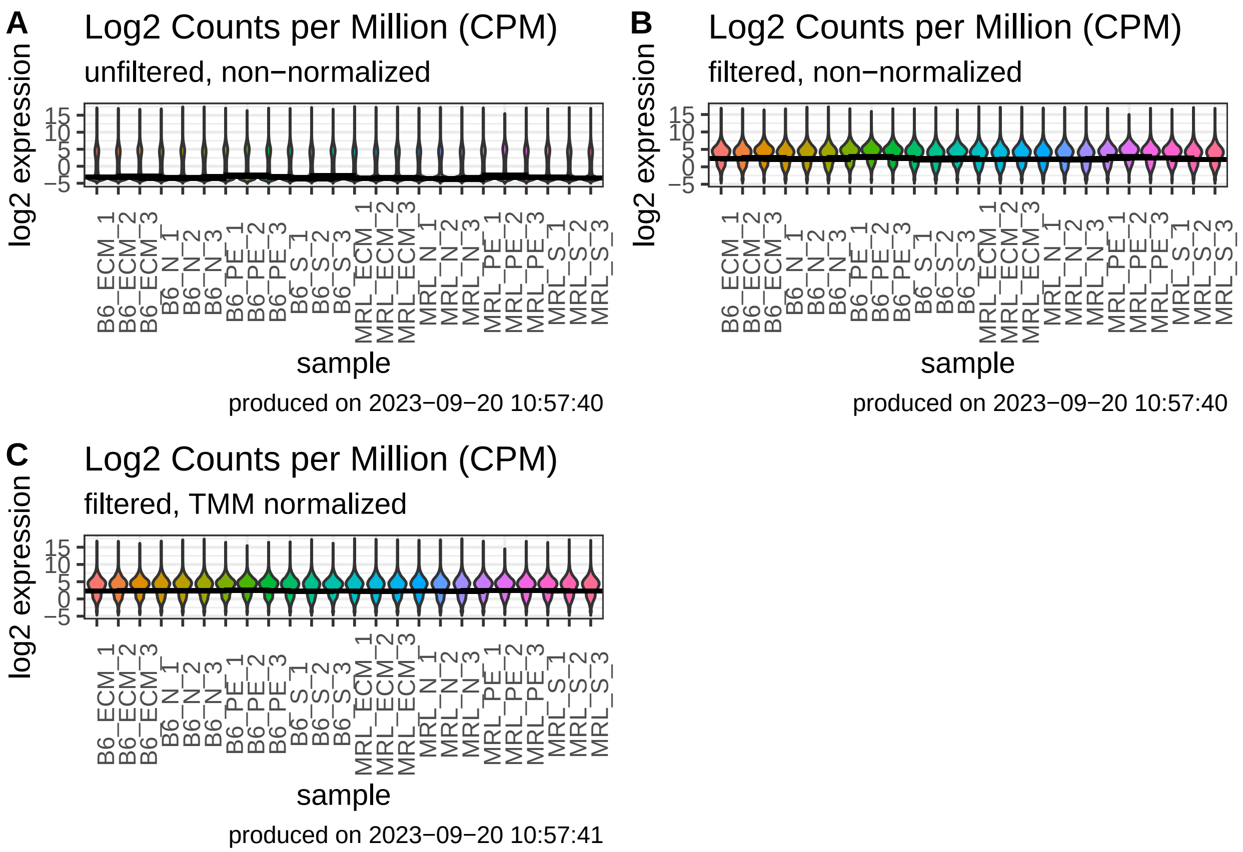

6 Data Analysis

6.1 PCA plot

```
group <- targets$group
group <- factor(group)

pca.res <- prcomp(t(log2.cpm.filtered.norm), scale.=F, retx=T)
pc.var <- pca.res$sdev^2
pc.per <- round(pc.var/sum(pc.var)*100, 1)
pca.res.df <- as_tibble(pca.res$x)
 pca.plot <- ggplot(pca.res.df) +
  aes(x=PC1, y=PC2, label=sampleLabels, color = group) +
  geom_point(size=4) +
  stat_ellipse() +
  xlab(paste0("PC1 (",pc.per[1],"%",")")) +
  ylab(paste0("PC2 (",pc.per[2],"%",")")) +
  labs(title="PCA plot",
       caption=paste0("produced on ", Sys.time())) +
  coord_fixed() +
```

```
  theme_bw()
pca.plot
```

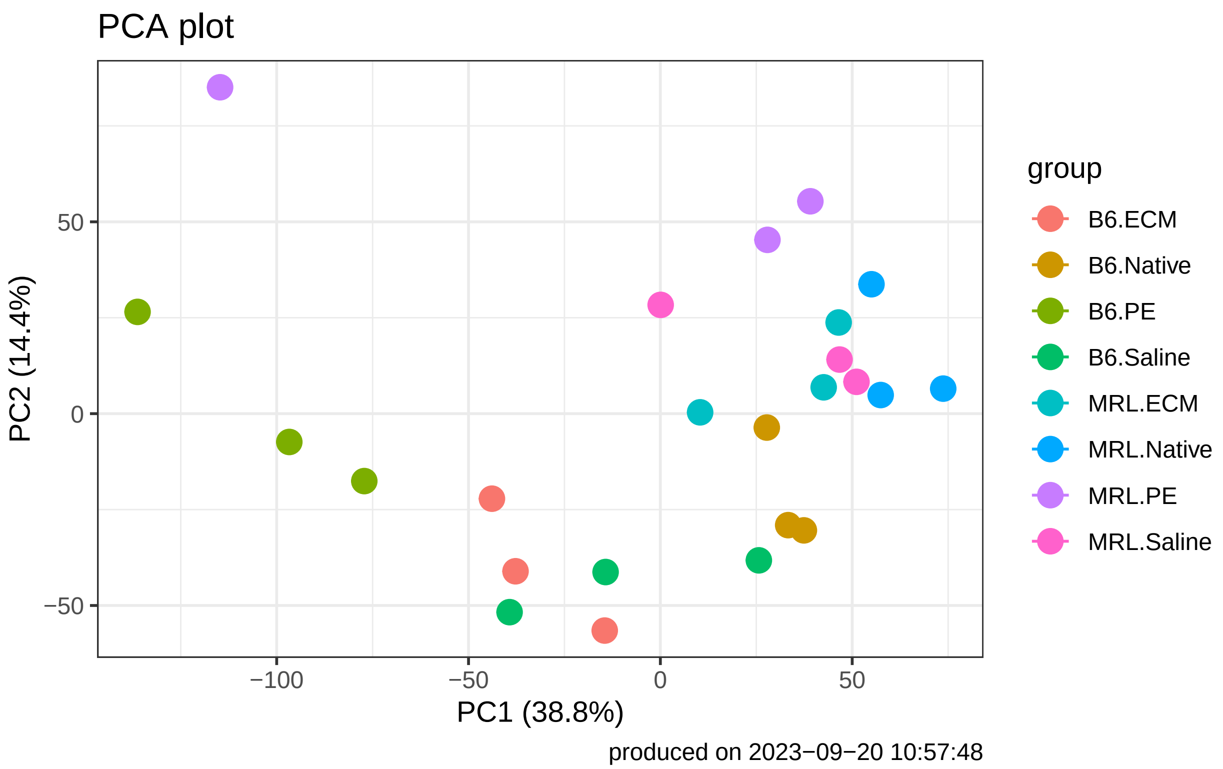

6.2 Volcano plot- Differential expression analysis: B6 vs MRL Saline

```
group <- factor(targets$group)
design <- model.matrix(~0 + group)
colnames(design) <- levels(group)

v.DEGList.filtered.norm <- voom(myDGEList.filtered.norm, design, plot = TRUE)
```

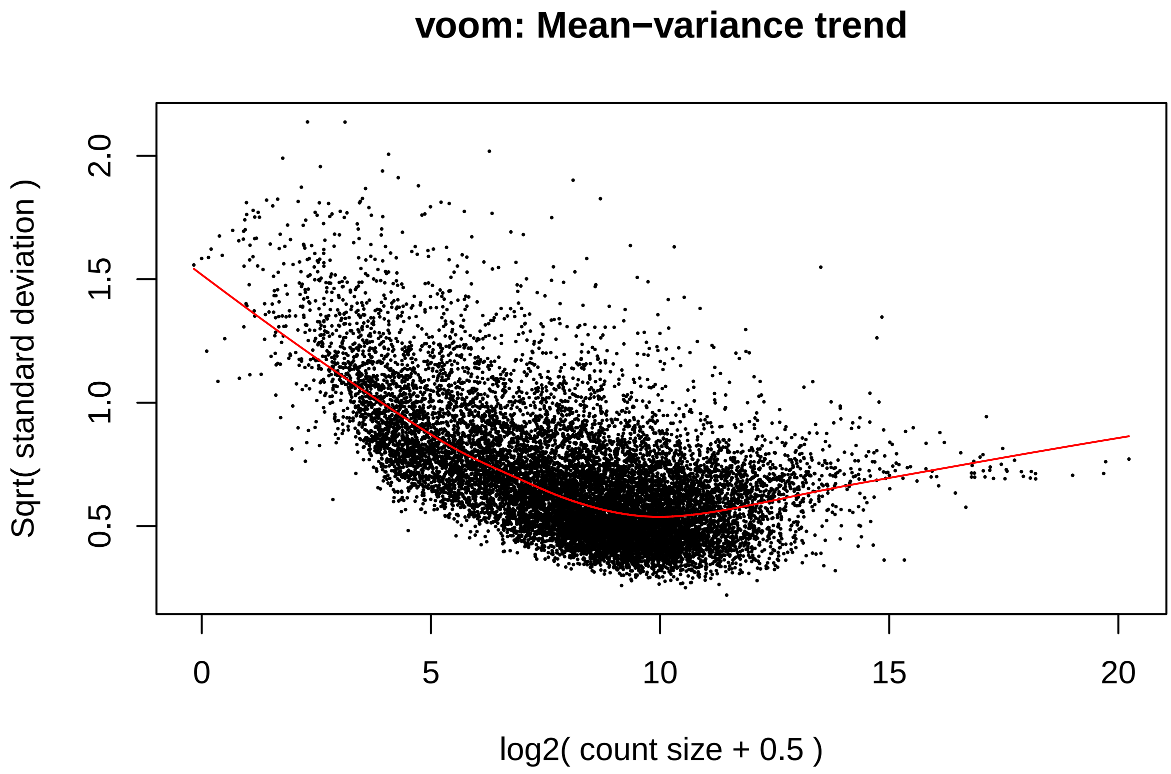


```
fit <- lmFit(v.DEGList.filtered.norm, design)
contrast.matrix <- makeContrasts(genotype1 = B6.Native - MRL.Native,
                                 genotype2 = B6.Saline - MRL.Saline,
                                 genotype3 = B6.PE - MRL.PE,
                                 genotype4 = B6.ECM - MRL.ECM,
                                 levels=design)
fits <- contrasts.fit(fit, contrast.matrix)
ebFit <- eBayes(fits)

myTopHits <- topTable(ebFit, adjust ="BH", coef=2, number=40000, sort.by="logFC")
myTopHits.df <- myTopHits %>%
  as_tibble(rownames = "geneID")

keyvals <- ifelse(
  myTopHits$logFC < -2, 'cornflowerblue',
  ifelse(myTopHits$logFC > 2, 'salmon',
         'black'))
keyvals[is.na(keyvals)] <- 'black'
names(keyvals)[keyvals == 'salmon'] <- 'high'
names(keyvals)[keyvals == 'black'] <- 'mid'
names(keyvals)[keyvals == 'cornflowerblue'] <- 'low'

EnhancedVolcano(myTopHits.df,
                lab = rownames(myTopHits),
                x = 'logFC',
                y='adj.P.Val',
```

```
                title = NULL,
                subtitle= NULL,
                caption = NULL,
                xlim = c(-11,11),
                ylim = c(0,12),
                xlab = bquote(~Log[2]~ 'fold change'),
                ylab = bquote(~-Log[10]~ 'FDR'),
                pCutoff = 0.01,
                FCcutoff = 2.0,
                pointSize = 5.0,
                labSize = 4.0,
                colAlpha = 1,
                colCustom = keyvals,
                cutoffLineType = 'blank',
                legendPosition = 'none',
                #legendLabSize = 14,
                #legendIconSize = 4.0,
                #colGradient = c('red3', 'royalblue'),
                drawConnectors = FALSE,
                #widthConnectors = 0.75,
gridlines.major = FALSE,
gridlines.minor = FALSE)
```

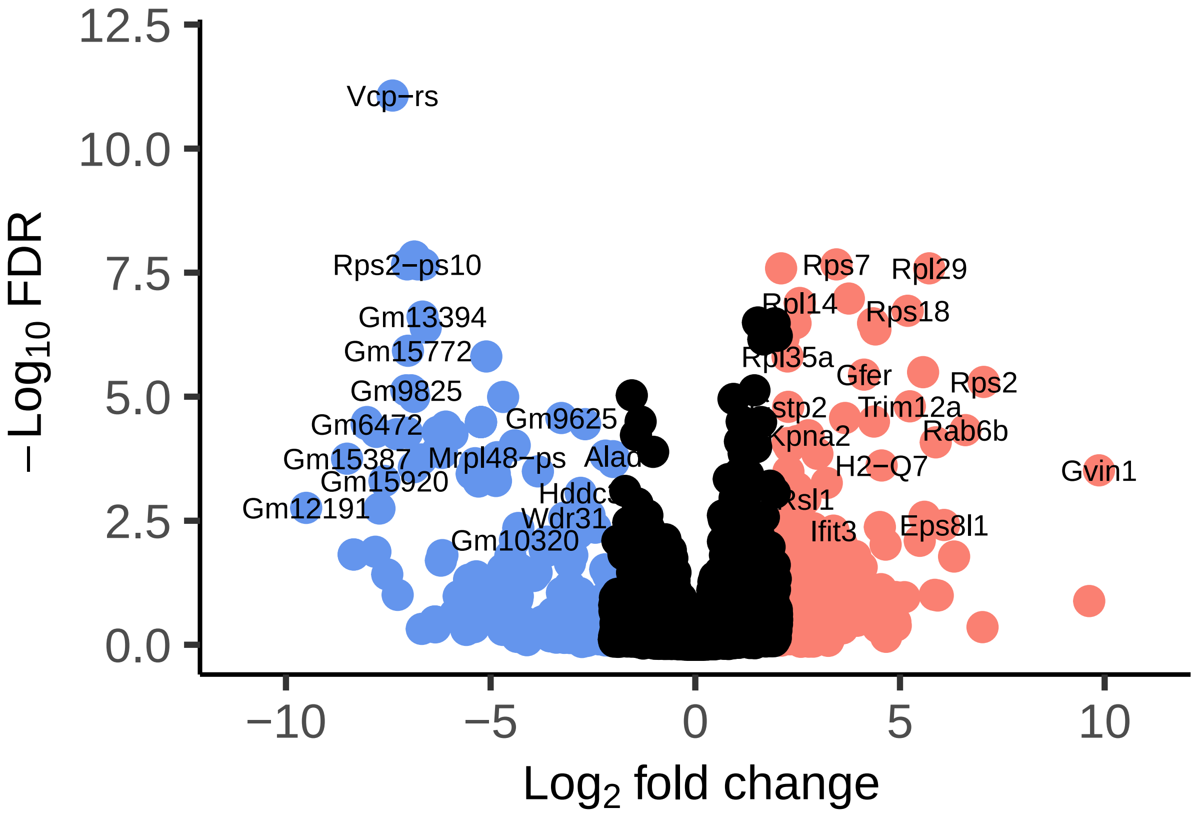

6.3 Heatmap: Top 50 differentially expressed genes- B6 vs MRL Uninjured

```
myheatcolors3 <- brewer.pal(name="RdYlBu", n=10)
results <- decideTests(ebFit, method="separate", adjust.method="BH", p.value=0.05, lfc=1)
colnames(v.DEGList.filtered.norm$E) <- sampleLabels
diffGenes <- v.DEGList.filtered.norm$E[results[,3] !=0,]
diffGenes2 <- diffGenes[1:50,c(4,5,6,16,17,18)]
pheatmap(diffGenes2,
          color= myheatcolors3, scale="row",cluster_rows = TRUE, cluster_cols = TRUE,
         clustering_distance_rows = "euclidean", clustering_distance_cols = "euclidean",
         clustering_method = "complete", show_rownames = TRUE, annotation_names_row = TRUE, fontsize_ro
```

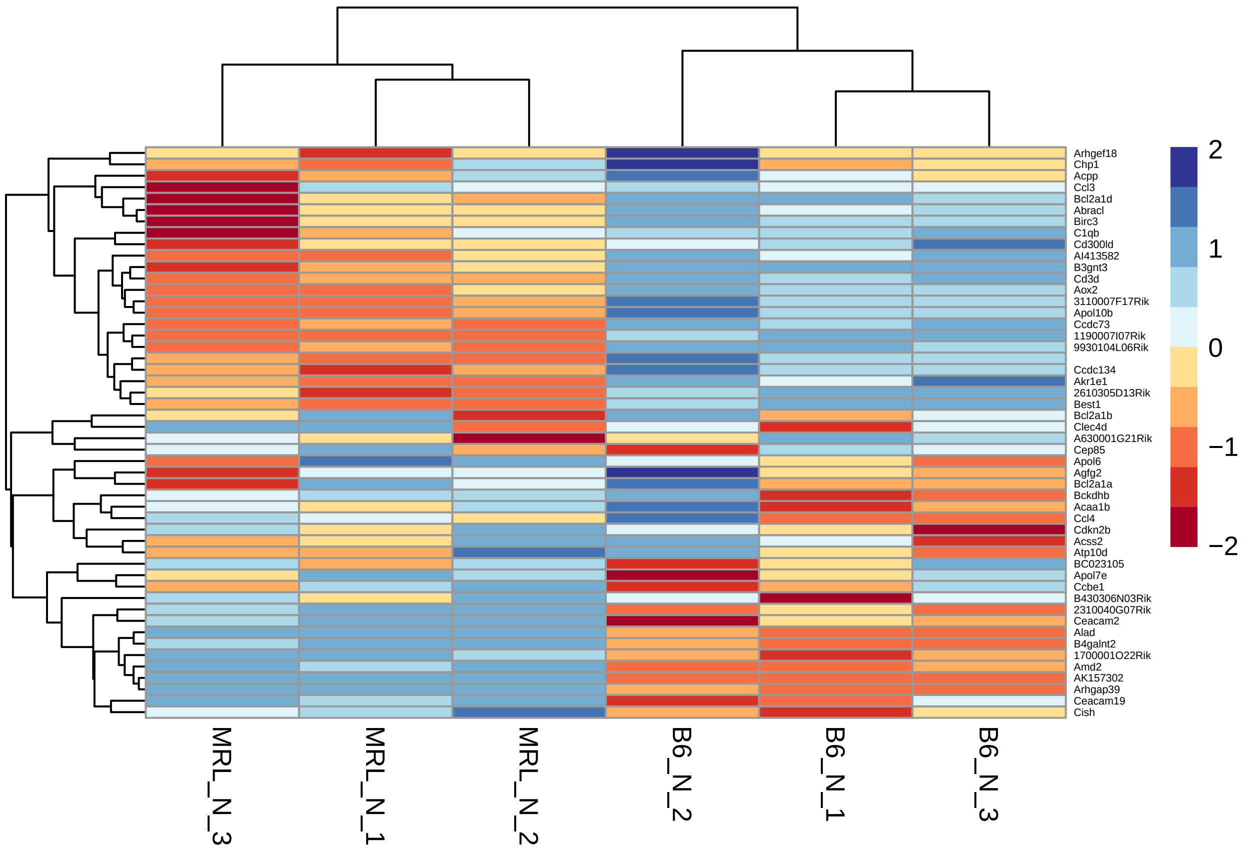

6.4 Gene Ontology and Pathway Assignment: Saline

```
myTopHits <- topTable(ebFit, adjust ="BH", coef=2, number=250, sort.by="logFC")
myTopHits.df <- myTopHits %>%
  as_tibble(rownames = "geneID")
gost.res <- gost(rownames(myTopHits), organism = "mmusculus", correction_method = "fdr")
gost1<- gostplot(gost.res, interactive = F, capped = F)

publish_gostplot(
  gost1,
```

```
  #highlight_terms = c("GO:"),
  filename = NULL,
  width = NA,
  height = NA)
```

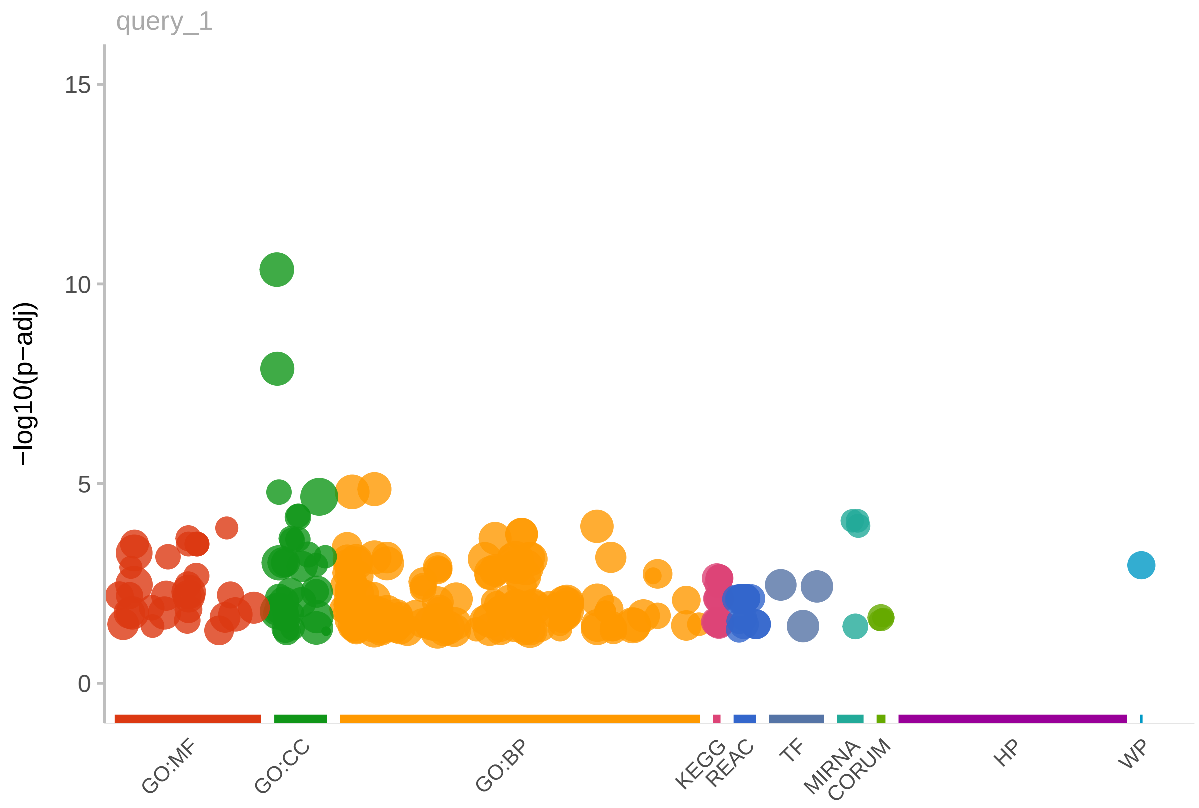

6.5 Gene Set Enrichment Analysis- B6 vs MRL: Saline

```
myTopHits.Saline <- topTable(ebFit, adjust ="BH", coef=2, number=5000, sort.by="logFC")
myTopHits.Saline.df <- myTopHits.Saline %>%
  as_tibble(rownames = "geneID")
hs_gsea <- msigdbr(species = "Mus musculus")
hs_gsea %>%
  dplyr::distinct(gs_cat, gs_subcat) %>%
  dplyr::arrange(gs_cat, gs_subcat)

hs_gsea_c2 <- msigdbr(species = "Mus musculus",
                      category = "C2") %>%
  dplyr::select(gs_name, gene_symbol)

mydata.Saline.df.sub <- dplyr::select(myTopHits.Saline.df, geneID, logFC)

mydata.gsea.Saline <- mydata.Saline.df.sub$logFC
names(mydata.gsea.Saline) <- as.character(mydata.Saline.df.sub$geneID)
```

```
mydata.gsea.Saline <- sort(mydata.gsea.Saline, decreasing = TRUE)

set.seed(1234)
myGSEA.res.Saline <- GSEA(mydata.gsea.Saline, TERM2GENE=hs_gsea_c2, verbose=FALSE, seed= TRUE)
myGSEA.Saline.df <- as_tibble(myGSEA.res.Saline@result)
```

```
GSEA1<- gseaplot2(myGSEA.res.Saline,
          geneSetID =14,
          base_size = 7,
          color = "blue",
          pvalue_table = FALSE)

GSEA2 <- gseaplot2(myGSEA.res.Saline,
          geneSetID =24,
          base_size = 7,
          color = "blue",
          pvalue_table = FALSE)

GSEA3 <- gseaplot2(myGSEA.res.Saline,
          geneSetID =34,
          base_size = 7,
          color = "blue",
          pvalue_table = FALSE)

plot_grid(GSEA1, GSEA2, GSEA3, labels = c('Ichiba-GVHD', 'Dauer-Stat3 targets', 'Sana- Response to IFNg
```

6.6 Gene Set Enrichment Analysis- B6 vs MRL: PE

```
myTopHits.PE <- topTable(ebFit, adjust ="BH", coef=3, number=5000, sort.by="logFC")
myTopHits.PE.df <- myTopHits.PE %>%
  as_tibble(rownames = "geneID")
hs_gsea <- msigdbr(species = "Mus musculus")
hs_gsea %>%
  dplyr::distinct(gs_cat, gs_subcat) %>%
  dplyr::arrange(gs_cat, gs_subcat)

hs_gsea_c2 <- msigdbr(species = "Mus musculus",
                      category = "C2") %>%
  dplyr::select(gs_name, gene_symbol)

mydata.PE.df.sub <- dplyr::select(myTopHits.PE.df, geneID, logFC)

mydata.gsea.PE <- mydata.PE.df.sub$logFC
names(mydata.gsea.PE) <- as.character(mydata.PE.df.sub$geneID)
mydata.gsea.PE <- sort(mydata.gsea.PE, decreasing = TRUE)

set.seed(1234)
myGSEA.res.PE <- GSEA(mydata.gsea.PE, TERM2GENE=hs_gsea_c2, verbose=FALSE, seed = TRUE)
myGSEA.PE.df <- as_tibble(myGSEA.res.PE@result)
```

```
myGSEA.res.PE

GSEA4 <- gseaplot2(myGSEA.res.PE,
          geneSetID =48,
          base_size = 7,
          color = "blue",
          pvalue_table = FALSE)

GSEA5 <- gseaplot2(myGSEA.res.PE,
          geneSetID =15,
          base_size = 7,
          color = "blue",
          pvalue_table = FALSE)

GSEA6 <- gseaplot2(myGSEA.res.PE,
          geneSetID =7,
          base_size = 7,
          color = "blue",
          pvalue_table = FALSE)

plot_grid(GSEA4, GSEA5, GSEA6, labels = c('Li-Adipogenesis by activated Pparg', 'Reactome-Neutrophil De


myGSEA.PE.df <- myGSEA.PE.df %>%
  mutate(phenotype = case_when(
    NES > 0 ~ "B6.PE",
    NES <= 0 ~ "MRL.PE"))


ggplot(myGSEA.PE.df[c(8,13,20,23,102,107,124,127,160,282),], aes(x=phenotype, y=ID)) +
  geom_point(aes(size=setSize, color = p.adjust)) +
  scale_color_gradient(low="blue", high="red") +
  #theme_calc() +
  theme(legend.position="right", axis.title = element_text(size = 10),
        panel.background = element_blank(),
        panel.border = element_blank(),
        panel.grid.major = element_blank(),
        panel.grid.minor = element_blank(), legend.text = element_text(size = 10),
        axis.text.x=element_text(size=13, colour = "black"),
        axis.text.y = element_text(size=8, colour = "black"),
        axis.ticks = element_blank())
```

6.7 ImmQuant scores and gene signatures correlation plot: PE

```
M <- read_tsv("Correlation plot data PE adipogenesis.txt")

M1 <- as_data_frame(M)
M2 <- cor(M1[sapply(M1, is.numeric)], method="spearman")

corrplot(M2, order ='AOE', type = 'lower', tl.col= 'black', diag= FALSE)
```

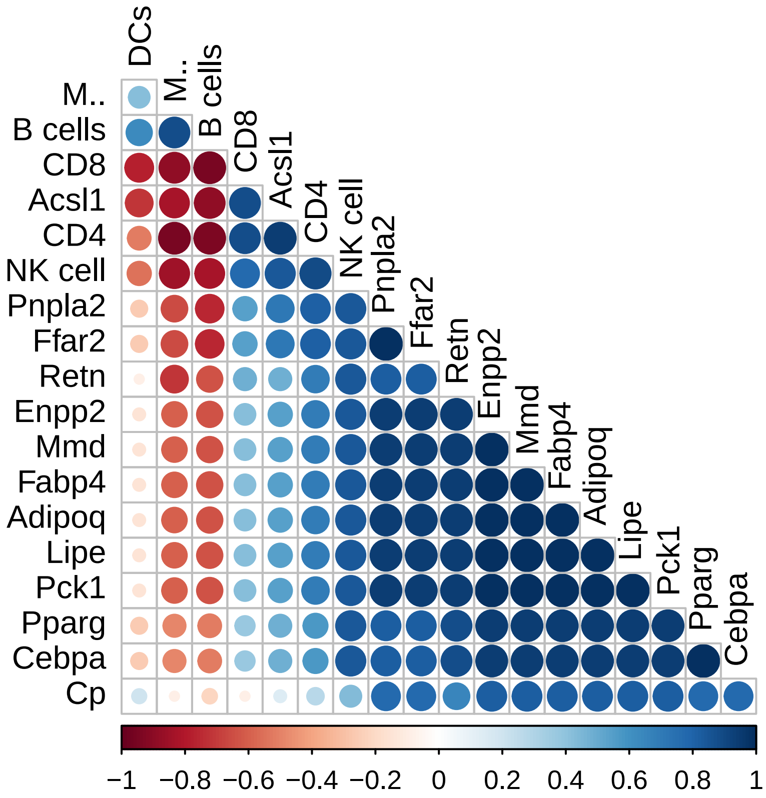

6.8 Heatmap of differentially expressed genes: ECM vs PE

```
myheatcolors2 <- colorRampPalette(colors=c("darkmagenta","black","gold"))(100)

mySelectedGenes <- read_tsv("Heatmap genes_PE_ECM.txt")

HM1 <- mySelectedGenes %>% remove_rownames %>% column_to_rownames(var="geneID")

HM1.matrix <- as.matrix(HM1)
heatmap.2(HM1.matrix,
          Rowv=FALSE, Colv=FALSE,
          col=myheatcolors2,
          scale="row", density.info="none",
          trace="none", labCol=NA,
          cexRow=0.75, cexCol=1, margins=c(8,20), key = T)
```

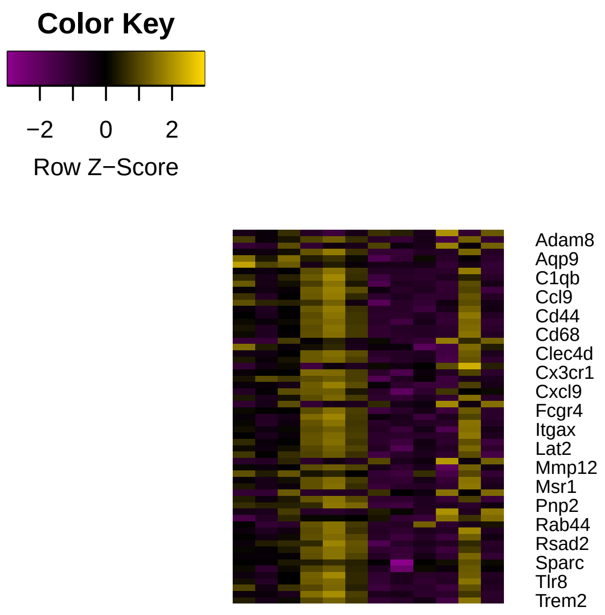

6.9 Gene Set Enrichment Analysis- B6 vs MRL: ECM

```
myTopHits.ECM <- topTable(ebFit, adjust ="BH", coef=4, number=5000, sort.by="logFC")
myTopHits.ECM.df <- myTopHits.ECM %>%
  as_tibble(rownames = "geneID")
hs_gsea <- msigdbr(species = "Mus musculus")
hs_gsea %>%
  dplyr::distinct(gs_cat, gs_subcat) %>%
  dplyr::arrange(gs_cat, gs_subcat)

hs_gsea_c2 <- msigdbr(species = "Mus musculus",
                      category = "C2") %>%
  dplyr::select(gs_name, gene_symbol)

mydata.df.ECM.sub <- dplyr::select(myTopHits.ECM.df, geneID, logFC)

mydata.gsea.ECM <- mydata.df.ECM.sub$logFC
names(mydata.gsea.ECM) <- as.character(mydata.df.ECM.sub$geneID)
mydata.gsea.ECM <- sort(mydata.gsea.ECM, decreasing = TRUE)

set.seed(1234)

myGSEA.res.ECM <- GSEA(mydata.gsea.ECM, TERM2GENE=hs_gsea_c2, verbose=FALSE, seed= TRUE)
myGSEA.ECM.df <- as_tibble(myGSEA.res.ECM@result)
```

```
myGSEA.res.ECM

GSEA7 <- gseaplot2(myGSEA.res.ECM,
          geneSetID =44,
          base_size = 7,
          color = "blue",
          pvalue_table = FALSE)

GSEA8 <- gseaplot2(myGSEA.res.ECM,
          geneSetID =86,
          base_size = 7,
          color = "blue",
          pvalue_table = FALSE)

GSEA9 <- gseaplot2(myGSEA.res.ECM,
          geneSetID =90,
          base_size = 7,
          color = "blue",
          pvalue_table = FALSE)

plot_grid(GSEA7, GSEA8, GSEA9, labels = c('Naba-Matrisome', 'KEGG-Asthma', 'Rutella- Response to HGF Up


myGSEA.ECM.df <- myGSEA.ECM.df %>%
  mutate(phenotype = case_when(
    NES > 0 ~ "B6.ECM",
    NES <= 0 ~ "MRL.ECM"))


ggplot(myGSEA.ECM.df[c(22,38,40,60,67,68,76,83,91,102,92),], aes(x=phenotype, y=ID)) +
  geom_point(aes(size=setSize, color = p.adjust)) +
  scale_color_gradient(low="blue", high="red") +
  #theme_calc() +
  theme(legend.position="right", axis.title = element_text(size = 10),
        panel.background = element_blank(),
        panel.border = element_blank(),
        panel.grid.major = element_blank(),
        panel.grid.minor = element_blank(), legend.text = element_text(size = 10),
        axis.text.x=element_text(size=13, colour = "black"),
        axis.text.y = element_text(size=8, colour = "black"),
        axis.ticks = element_blank())
```

6.10 ImmQuant scores and gene signatures correlation plot: ECM

```
M <- read_tsv("Correlation plot data ECM HGF.txt")

M1 <- as_data_frame(M)
M2 <- cor(M1[sapply(M1, is.numeric)], method="spearman")

corrplot(M2, order ='AOE', type = 'lower', tl.col= 'black', diag= FALSE)
```

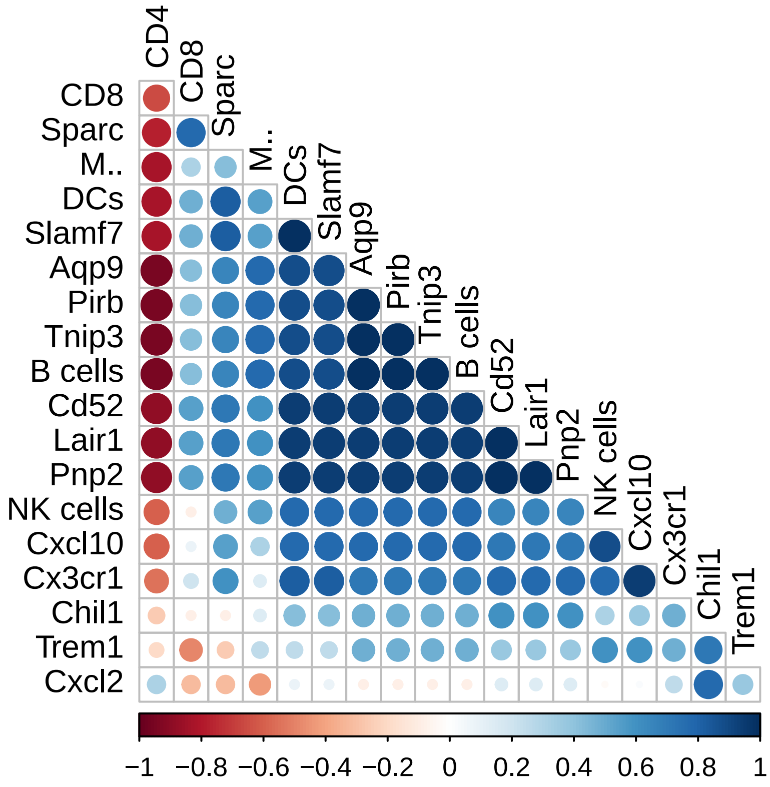

6.11 Interaction plot- PE: Immune Cell Targets and Adipogenesis

```
df <- read_tsv("PE interaction plot data.txt")
grid.col = c(Pparg = "grey",
             Acsl1 = "grey",
             Cebpa= "grey",
             Cp= "grey",
             Pnpla2= "grey",
             Mmd= "grey",
             Fabp4= "grey",
             Adipoq= "grey",
             Pck1= "grey",
             Adam8 = "skyblue2",
             Arg1 = "lightcoral",
             Bln = "skyblue2",
             Cd44 = "lightcoral",
             Cd53 = "lightcoral",
             Cd68= "lightcoral",
             Clec7a= "lightcoral",
             Cxcl2= "lightcoral",
             Il1b= "skyblue2",
             Itgam= "lightcoral",
             Itgax= "lightcoral",
             Itgb2= "skyblue2",
```

```
              Lat2 = "skyblue2",
              Rab44 ="skyblue2",
              Rac2 = "lightcoral",
              Tlr2= "skyblue2",
              Tlr8= "skyblue2",
              Trem2= "lightcoral")
circos.clear()
par(cex=1)
circos.par(gap.after = c("Pparg"=1, "Acsl1"=1, "Cebpa"=1, "Cp"=12, "Pnpla2"=1, "Mmd"=1, "Fabp4"=1, "Adip
                         "Lat2"=1, "Rab44"=1, "Tlr2"=1, "Tlr8"=12, "Arg1"=1, "Cd44"=1, "Cd53"=1, "Cd68"=
chordDiagram(df, grid.col = grid.col, annotationTrack = c("name", "grid"),
             order = c("Pparg", "Acsl1", "Cebpa", "Pnpla2", "Mmd", "Fabp4", "Adipoq", "Pck1", "Cp", "Ada
                       "Lat2", "Rab44", "Tlr2", "Tlr8", "Arg1", "Cd44", "Cd53", "Cd68", "Clec7a", "Cxcl
```

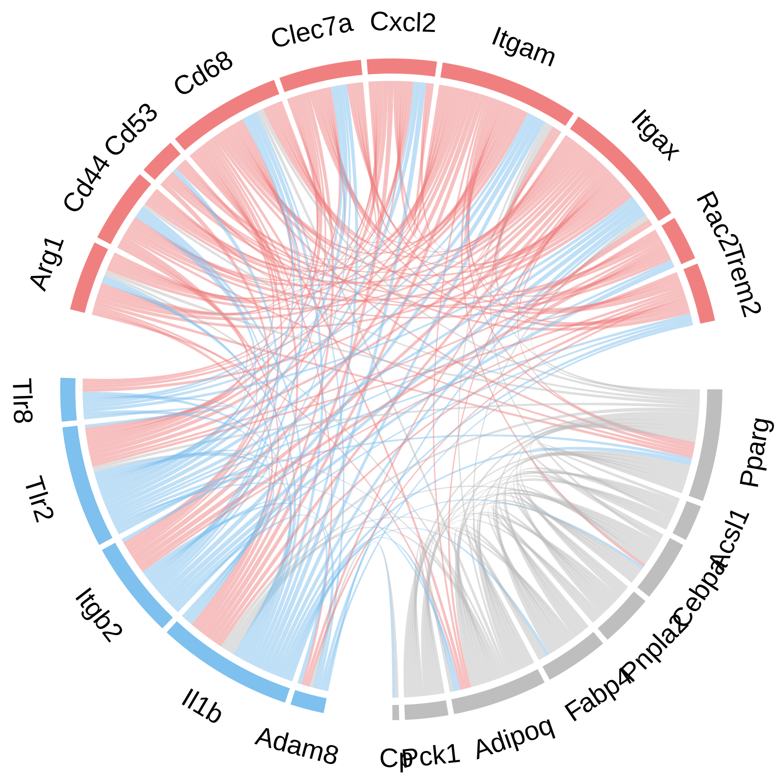

6.12 Interaction plot- ECM:HGF response and Eosinophil gene set

```
df2 <- read_tsv("ECM interactions plot4.txt")


grid.col2 = c(Mmp13="mediumpurple2",
Mmp12="mediumpurple2",
Adam8="mediumpurple2",
Cxcl2="mediumpurple2",
```

```
Cxcl10="mediumpurple2",
Col8a2="forestgreen",
Col23a1="forestgreen",
Col16a1="forestgreen",
Col6a5="forestgreen",
Col1a1="forestgreen",
Col4a3="forestgreen",
Col8a1="forestgreen",
Prg4="thistle3",
Cd40="thistle3",
Cd80="thistle3",
Lair1="thistle3",
Chil1="thistle3",
Cd84="thistle3")

circos.clear()
par(cex=1)

circos.par(gap.after = c("Mmp13"=1,
"Mmp12"=1,
"Adam8"=1,
"Cxcl2"=1,
"Cxcl10"=12,
"Col8a2"=1,
"Col23a1"=1,
"Col16a1"=1,
"Col6a5"=1,
"Col1a1"=1,
"Col4a3"=1,
"Col8a1"=12,
"Prg4"=1,
"Cd40"=1,
"Cd80"=1,
"Lair1"=1,
"Chil1"=1,
"Cd84"=12))

chordDiagram(df2, grid.col = grid.col2, annotationTrack = c("name", "grid"),
             order = c("Mmp13",
"Mmp12",
"Adam8",
"Cxcl2",
"Cxcl10",
"Col8a2",
"Col23a1",
"Col16a1",
"Col6a5",
"Col1a1",
"Col4a3",
"Col8a1",
"Prg4",
"Cd40",
"Cd80",
```

```
"Lair1",
"Chil1",
"Cd84"))
```

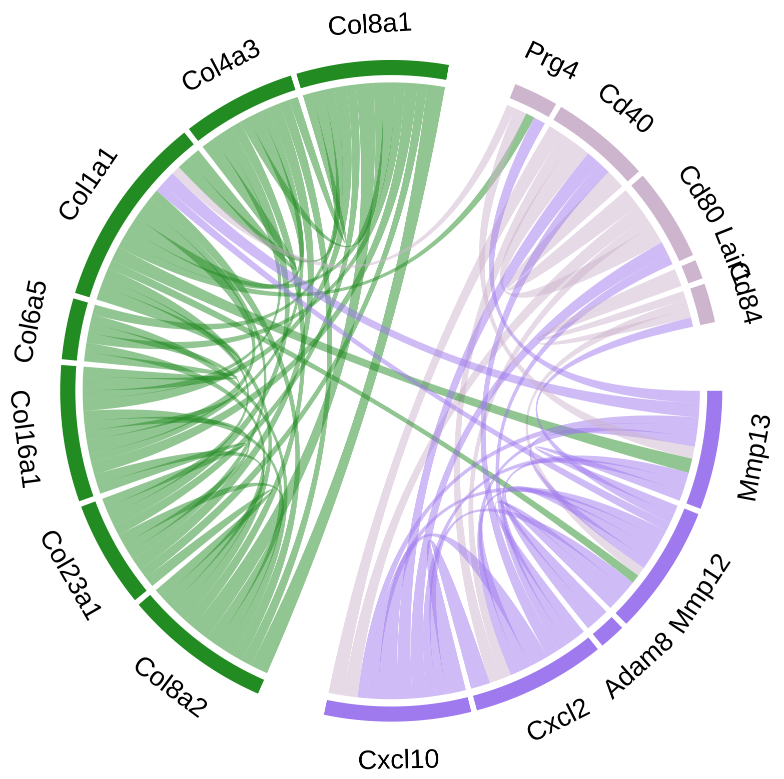

6.13 Ternary plot: M1 vs M2 : Coates macrophage gene set

```
library(ggtern)

Data.M1M2.MRL <- read_tsv("M1M2_MRL_ternary.txt")

Data.M1M2.MRL <- subset(Data.M1M2.MRL, !rowSums(Data.M1M2.MRL < 0))

plot11 <- ggtern(data = Data.M1M2.MRL,
                 aes(ECM, PE, Control))+
  stat_density_tern(geom = 'polygon',
                    n         = 205,
                    bdl = 0.020,
                    contour = TRUE,
                    aes(fill  = ..level..,
                        alpha = ..level..)) +
  geom_point(size=2, alpha= 0.5) +

  theme_rgbw(base_size = 14) +
```

```
  scale_fill_gradient(low = "blue",high = "red")  +
  guides(color = "none", fill = "none", alpha= "none")


plot11_tern <- plot11 + geom_text(data=~subset(.,geneID %in% c("Arg1","Tjp2","Uchl1", "Chil3", "Ada","Ma
                  aes(label=geneID),hjust=-0.2 , vjust=0.2 ,size=5)+

  theme_nomask()

Data.M1M2.B6 <- read_tsv("M1M2_B6_ternary.txt")

Data.M1M2.B6 <- subset(Data.M1M2.B6, !rowSums(Data.M1M2.B6 < 0))

plot12 <- ggtern(data = Data.M1M2.B6,
                 aes(ECM, PE, Control))+
  stat_density_tern(geom = 'polygon',
                    n         = 205,
                    bdl = 0.020,
                    contour = TRUE,
                    aes(fill  = ..level..,
                        alpha = ..level..)) +
  geom_point(size=2, alpha=0.5) +

  theme_rgbw(base_size = 14) +
  scale_fill_gradient(low = "blue",high = "red")  +
  guides(color = "none", fill = "none", alpha= "none")


plot12_tern <- plot12 + geom_text(data=~subset(.,geneID %in% c("Arg1","Tjp2","Uchl1", "Chil3", "Ada","Ma
                  aes(label=geneID),hjust=-0.2 , vjust=0.2 ,size=5)+

  theme_nomask()


plot11_tern
```

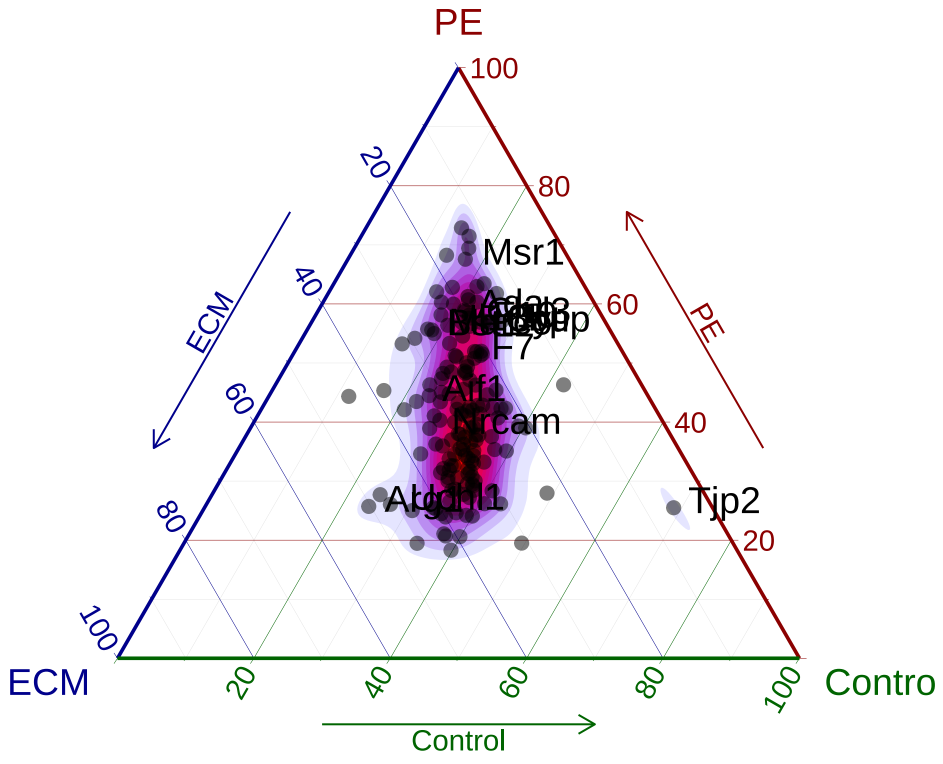
PE
100
80
60
40
20
ECM
20
40
60
80
100
ECM
Contro
Control
PE
Msr1
F7
Aif1
Tjp2

```
plot12_tern
```

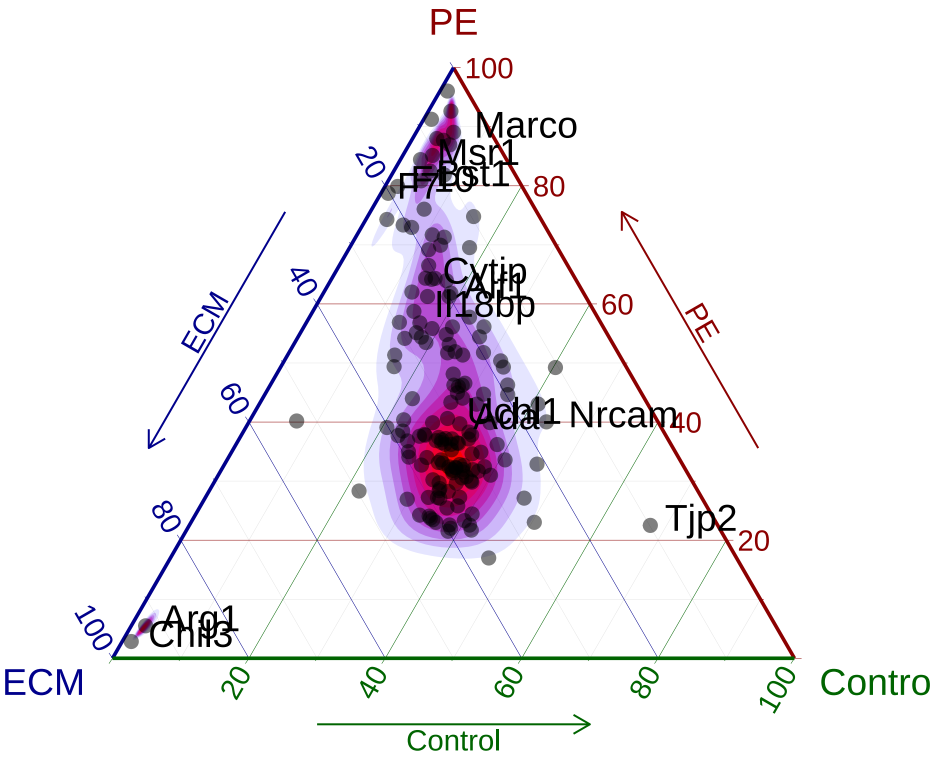

6.14 Ternary plot: Neutrophil gene set

```
Data.Neuts.B6 <- read_tsv("Neuts_B6_ternary2.txt")
plot13 <- ggtern(data = Data.Neuts.B6,
                 aes(ECM, PE, Control))+
  stat_density_tern(geom = 'polygon',
                    n         = 205,
                    bdl = 0.020,
                    contour = TRUE,
                    aes(fill  = ..level..,
                        alpha = ..level..)) +
  geom_point(size=2, alpha=0.5) +

  theme_rgbw(base_size = 14) +
  scale_fill_gradient(low = "blue",high = "red")  +
  guides(color = "none", fill = "none", alpha = "none")

plot13_tern <- plot13 +
  geom_text(data = ~subset(., geneID %in% c("Ccl9", "Ccr2", "Ccl8", "Ccl12", "Ctsb", "Adam8")), size =5
            aes(label = geneID, hjust = -0.1, vjust = -0.1)) +
  geom_text(data = ~subset(., geneID %in% c("Cxcl2", "Cybb", "Icam1", "Mmp9", "Myd88")), size =5,
            aes(label = geneID, hjust = 0.2, vjust = 0.4)) +
  geom_text(data = ~subset(., geneID %in% c("Ccl25")), size =5,
            aes(label = geneID,hjust = 0.5, vjust =0.5)) +
```

```
  theme_nomask()

Data.Neuts.MRL <- read_tsv("Neuts_MRL_ternary2.txt")
plot14 <- ggtern(data = Data.Neuts.MRL,
                 aes(ECM, PE, Control))+
  stat_density_tern(geom = 'polygon',
                    n         = 205,
                    bdl = 0.020,
                    contour = TRUE,
                    aes(fill  = ..level..,
                        alpha = ..level..)) +
  geom_point(size=2, alpha=0.5) +

  theme_rgbw(base_size = 14) +
  scale_fill_gradient(low = "blue",high = "red")  +
  guides(color = "none", fill = "none", alpha = "none")

plot14_tern <- plot14 +
  geom_text(data = ~subset(., geneID %in% c("Ccl9", "Ccr2", "Ccl8", "Ccl12", "Ctsb", "Adam8")), size =5
            aes(label = geneID, hjust = -0.1, vjust = -0.1)) +
  geom_text(data = ~subset(., geneID %in% c("Cxcl2", "Cybb", "Icam1", "Mmp9", "Myd88")), size =5,
            aes(label = geneID, hjust = 0.2, vjust = 0.4)) +
  geom_text(data = ~subset(., geneID %in% c("Ccl25")), size =5,
            aes(label = geneID,hjust = 0.5, vjust =0.5)) +
  theme_nomask()

plot13_tern
```

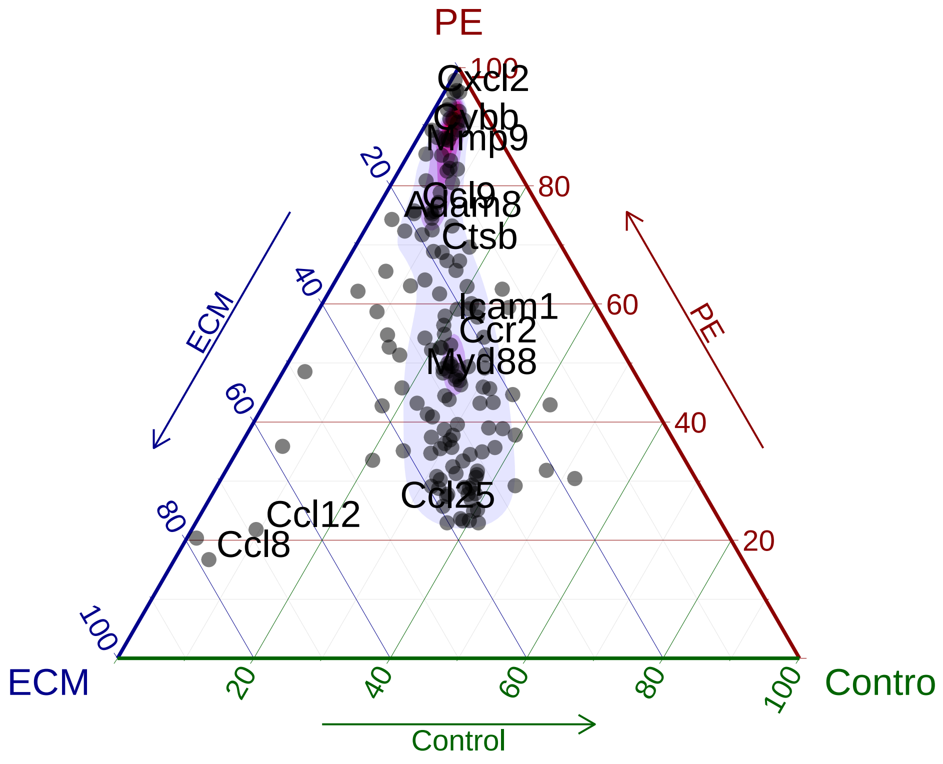
PE
Cxcl2
Cybb
Mmp9
Ccl9
Adam8
Ctsb
Icam1
Ccr2
Myd88
Ccl25
Ccl12
Ccl8
ECM
Contro
Control
PE
ECM
100
80
60
40
20
20
40
60
80
100
20
40
60
80
100

```
plot14_tern
```

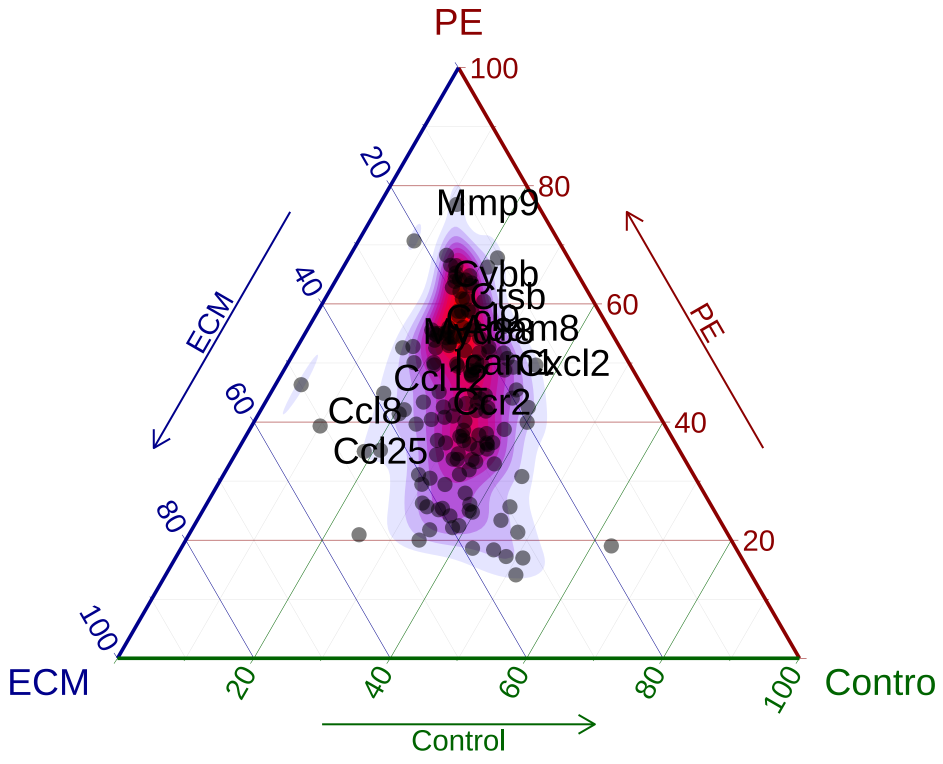

6.15 Ternary plot: Eosinophils gene set

```
Data.Eos.B6 <- read_tsv("Eos_B6_ternary.txt")
plot15 <- ggtern(data = Data.Eos.B6,
                 aes(ECM, PE, Control))+
  stat_density_tern(geom = 'polygon',
                    n        = 205,
                    bdl = 0.020,
                    contour = TRUE,
                    aes(fill  = ..level..,
                        alpha = ..level..)) +
  geom_point(size=2, alpha=0.5) +

  theme_rgbw(base_size = 14) +
  scale_fill_gradient(low = "blue",high = "red")  +
  guides(color = "none", fill = "none", alpha = "none")

plot15_tern <- plot15 +
  geom_text(data = ~subset(., geneID %in% c("Ccl12", "Ccl8", "Ccr2", "F2rl1", "Cd300a", "Adam8")), size
            aes(label = geneID, hjust = -0.1, vjust = -0.1)) +
  geom_text(data = ~subset(., geneID %in% c("Gata2", "Ccl9", "Ptger2", "Ccl2", "Cx3cl1")), size =5,
            aes(label = geneID, hjust = 0.2, vjust = 0.4)) +
  geom_text(data = ~subset(., geneID %in% c("Ccl25")), size =5,
            aes(label = geneID,hjust = 0.5, vjust =0.5)) +
```

```
  theme_nomask()


Data.Eos.MRL <- read_tsv("Eos_MRL_ternary.txt")
plot16 <- ggtern(data = Data.Eos.MRL,
                 aes(ECM, PE, Control))+
  stat_density_tern(geom = 'polygon',
                    n         = 205,
                    bdl = 0.020,
                    contour = TRUE,
                    aes(fill  = ..level..,
                        alpha = ..level..)) +
  geom_point(size=2, alpha=0.5) +

  theme_rgbw(base_size = 14) +
  scale_fill_gradient(low = "blue",high = "red")  +
  guides(color = "none", fill = "none", alpha = "none")

plot16_tern <- plot16 +
  geom_text(data = ~subset(., geneID %in% c("Ccl12", "Ccl8", "Ccr2", "F2rl1", "Cd300a", "Adam8")), size
            aes(label = geneID, hjust = -0.1, vjust = -0.1)) +
  geom_text(data = ~subset(., geneID %in% c("Gata2", "Ccl9", "Ptger2", "Ccl2", "Cx3cl1")), size =5,
            aes(label = geneID, hjust = 0.2, vjust = 0.4)) +
  geom_text(data = ~subset(., geneID %in% c("Ccl25")), size =5,
            aes(label = geneID,hjust = 0.5, vjust =0.5)) +
  theme_nomask()

plot15_tern
```

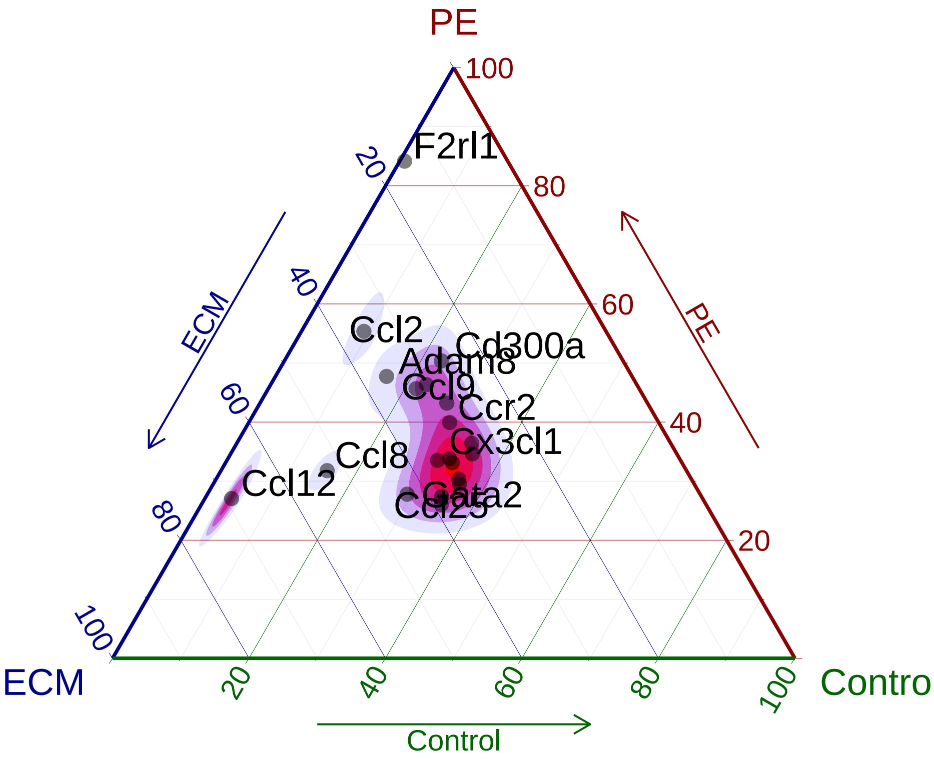
PE
100
80
60
40
20
F2rl1
Ccl2
Cd300a
Adam8
Ccl9
Ccr2
Cx3cl1
Ccl8
Ccl12
Gata2
Ccl25
ECM
20
40
60
80
100
PE
ECM
20
40
60
80
100
Contro
Control

```
plot16_tern
```

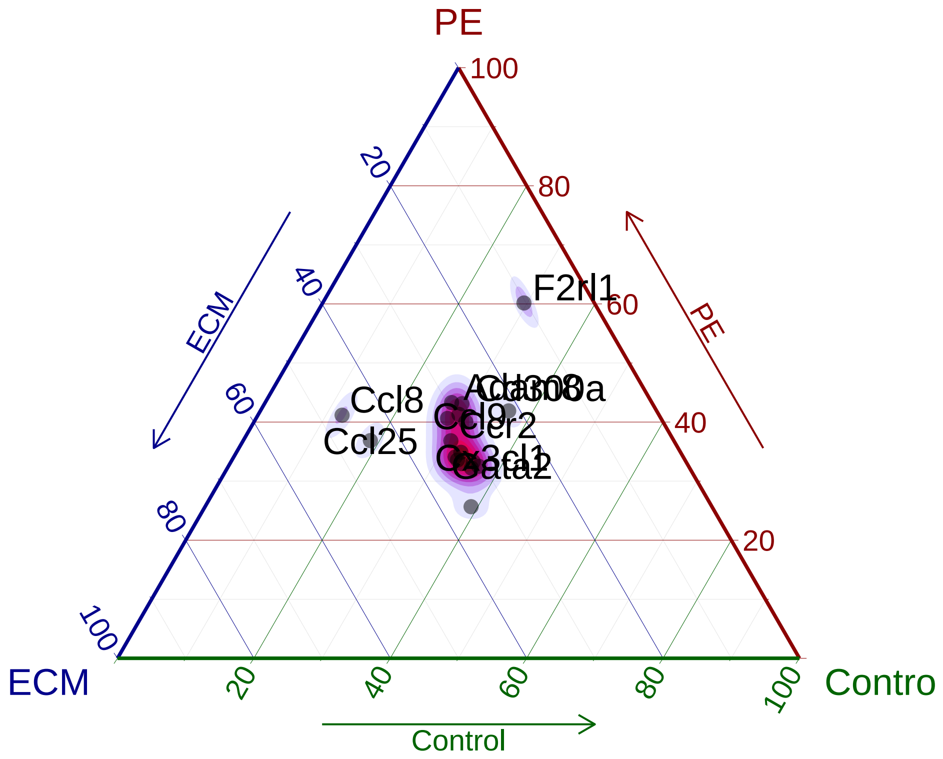
PE
100
80
60
40
20
ECM
Contro
Control
F2rl1
Ccl8
Ccl25
Ccl9
Ccr2
Gata2
